# Supplementary material for: Anti-Mycobacterial N-(2-Arylethyl)quinolin-3-amines Inspired by Marine Sponge-Derived Alkaloid
Source: Molecules. 2022 Dec 8;27(24):8701. doi: 10.3390/molecules27248701 (PMC9781020; doi:10.3390/molecules27248701)

molecules

MDPI

Supplementary Materials

Anti-mycobacterial *N*-(2-Arylethyl)quinolin-3-amines Inspired by Marine Sponge-Derived Alkaloid

Junya Mukomura [1], Hiroki Nonaka [2], Hiromasa Sato [2], Maho Kishimoto [1], Masayoshi Arai [2] and Naoyuki Kotoku [1,2,*]

[1] College of Pharmaceutical Sciences, Ritsumeikan University, 1-1-1 Noji-Higashi, Kusatsu, Shiga 525-8577, Japan; ph0107ir@ed.ritsumei.ac.jp (J.M.); ph0077hr@ed.ritsumei.ac.jp (M.K.)
[2] Graduate School of Pharmaceutical Sciences, Osaka University, 1-6 Yamadaoka, Suita, Osaka 565-0871, Japan; hiroki.olympique.lyonnais@gmail.com (H.N.); sato0704hiromasa@gmail.com (H.S.); araim@phs.osaka-u.ac.jp (M.A.)
* Correspondence: kotoku@fc.ritsumei.ac.jp; Tel.: +81-77-561-4920

Contents

^{1}H- and ^{13}C-NMR spectra for new compounds: pages S2-S21.

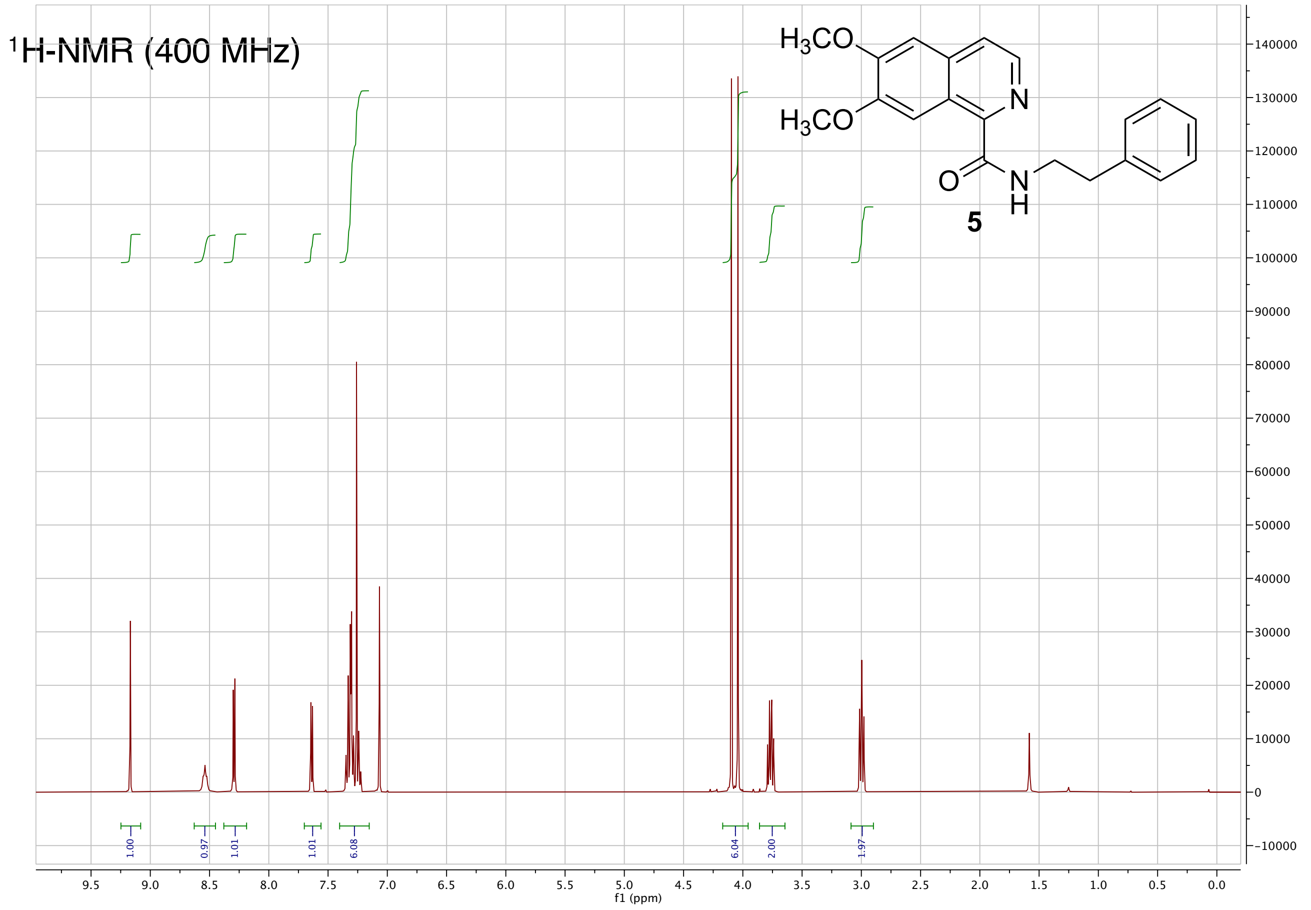

1H-NMR (400 MHz)
H3CO
H3CO
N
O
N
H
5
1.00
0.97
1.01
1.01
6.08
6.04
2.00
1.97
f1 (ppm)

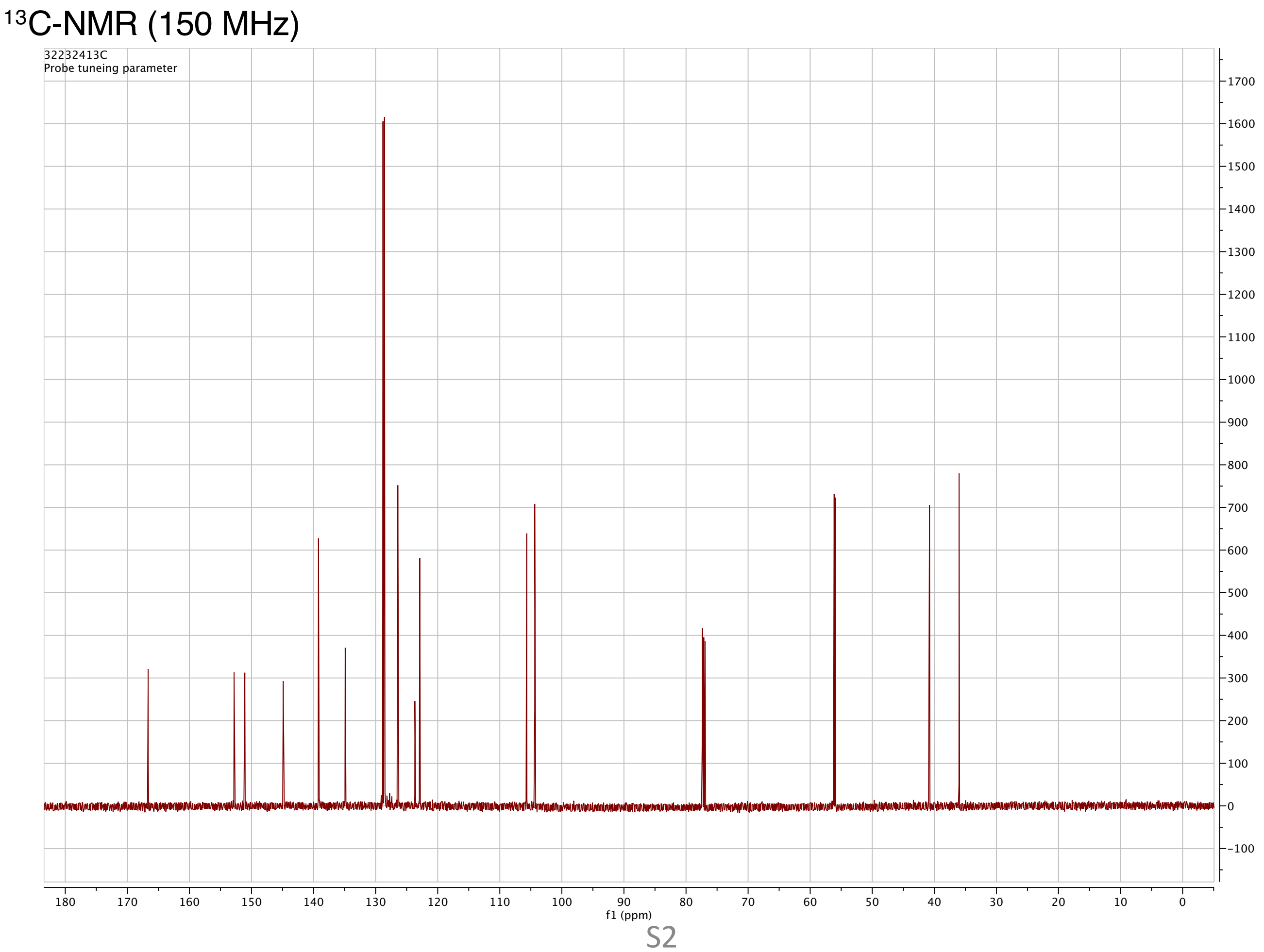

13C-NMR (150 MHz)
32232413C
Probe tuneing parameter
f1 (ppm)

^{1}H-NMR (500 MHz)

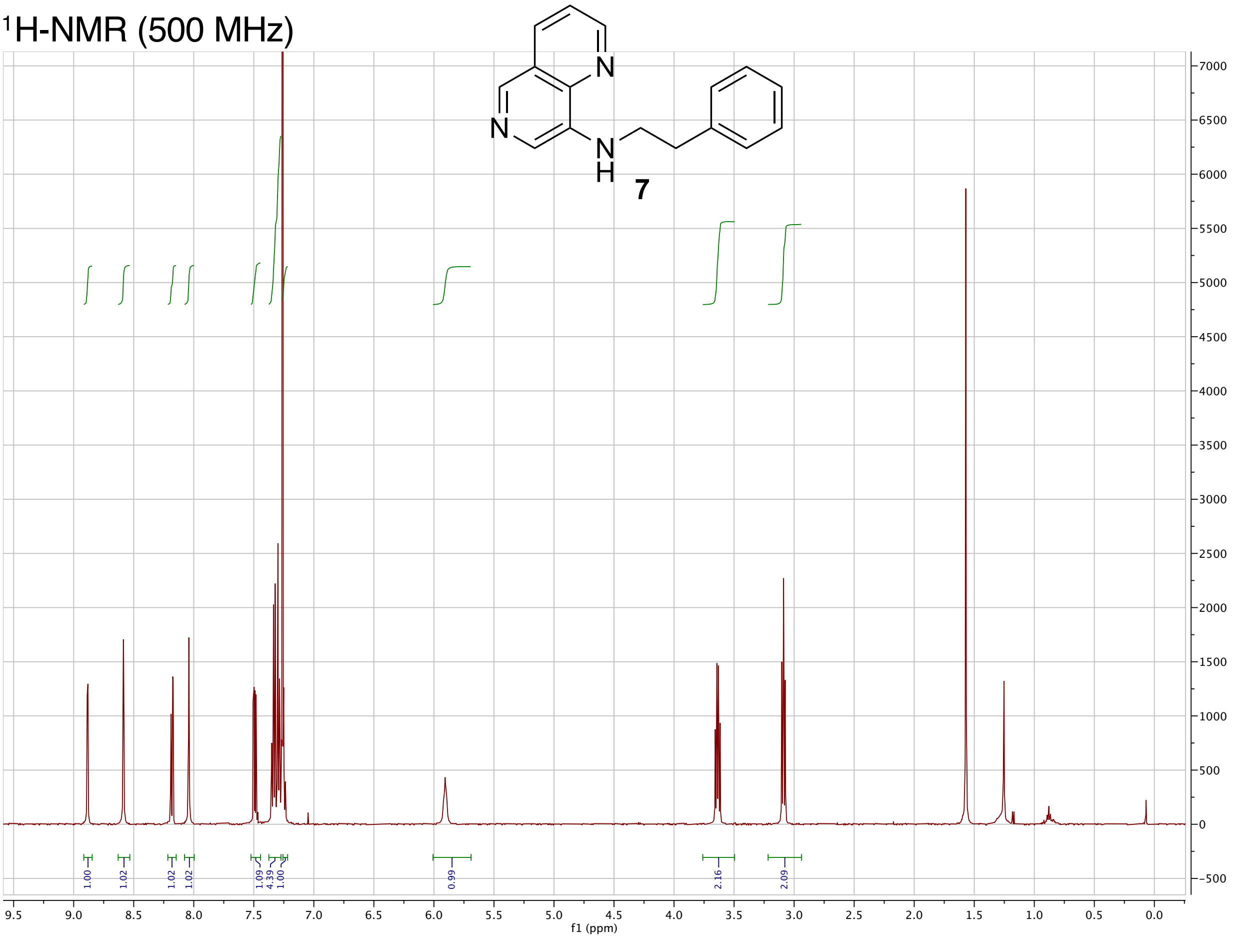

^{13}C-NMR (150 MHz)

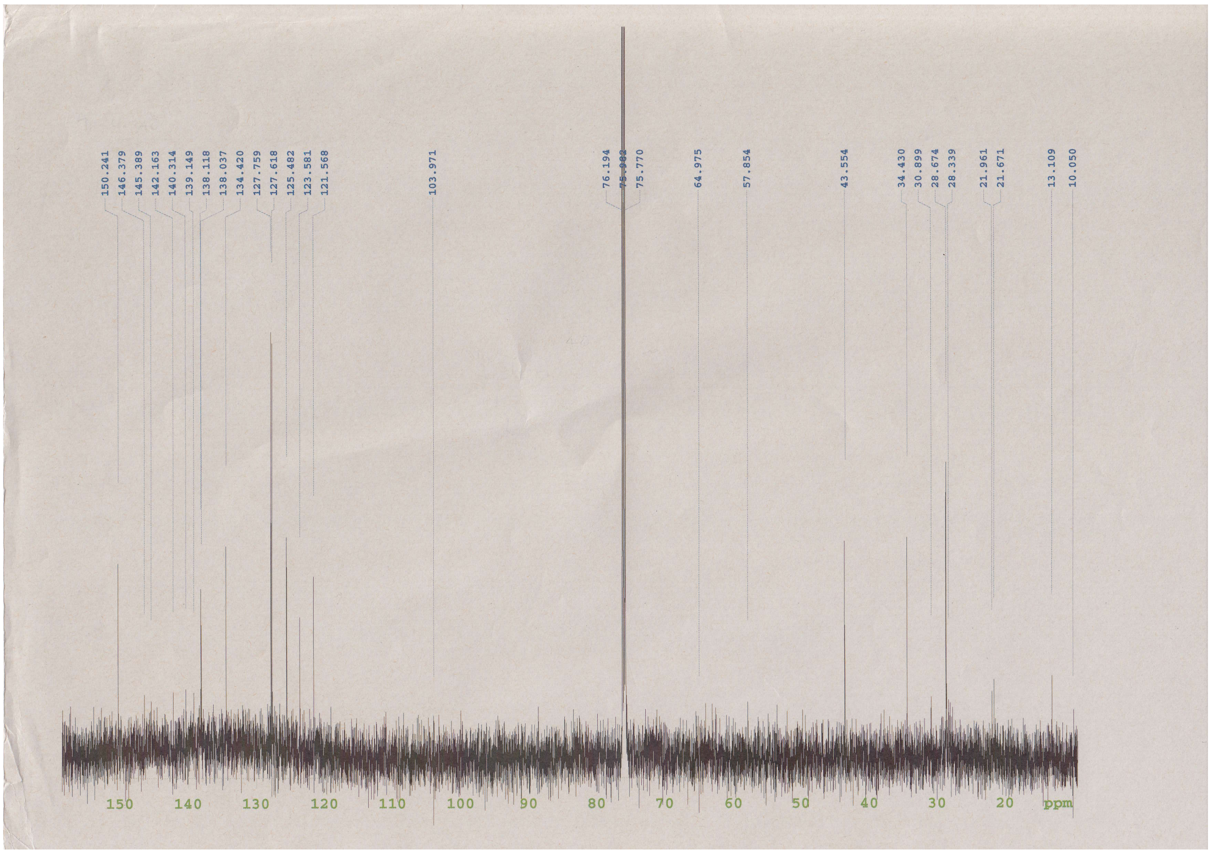

^{1}H-NMR (400 MHz)

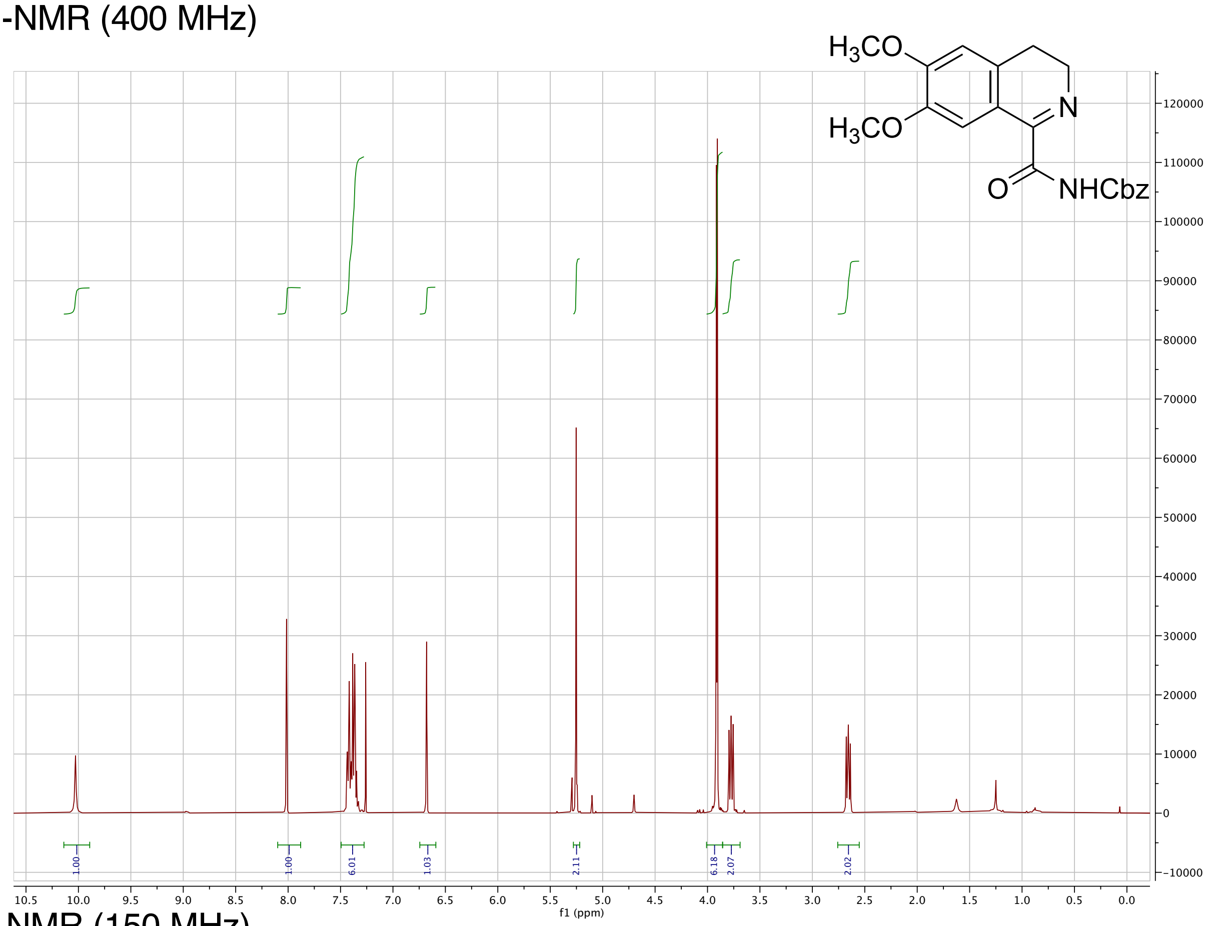

^{13}C-NMR (150 MHz)

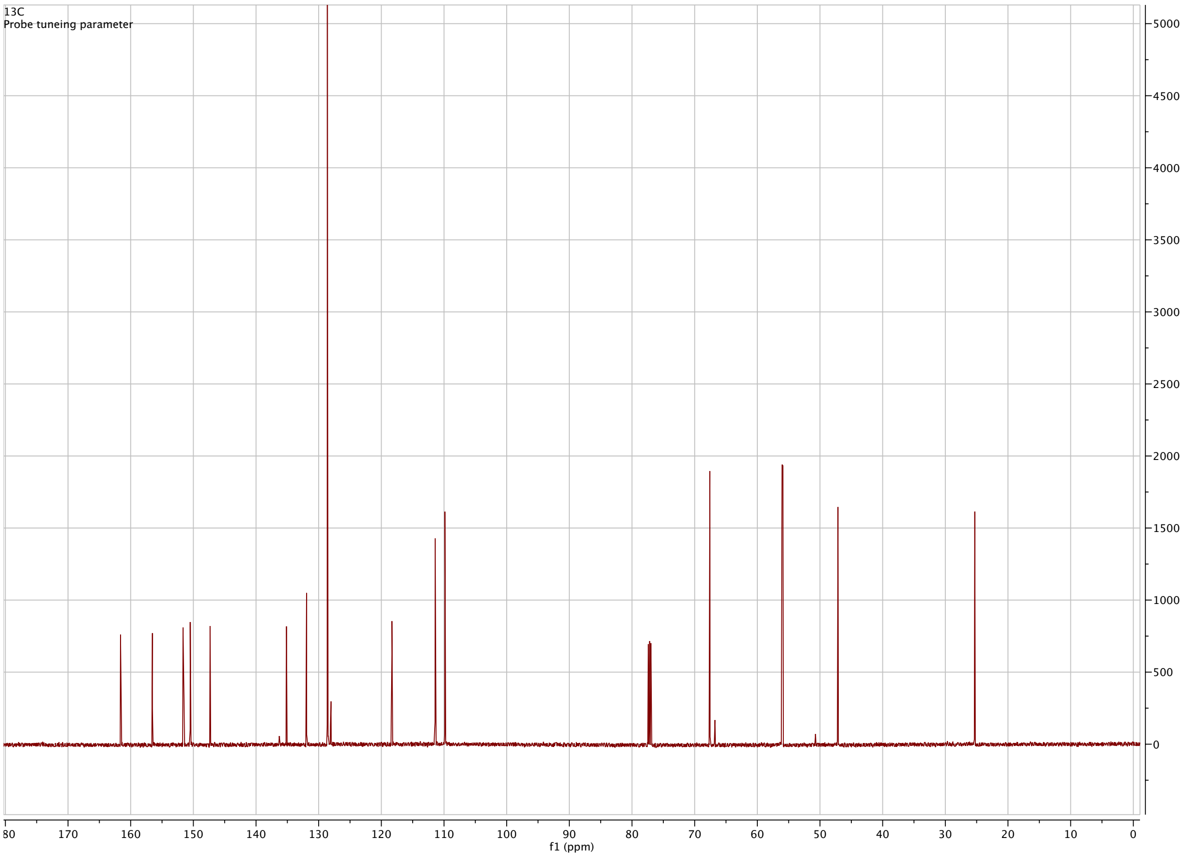

^{1}H-NMR (300 MHz)

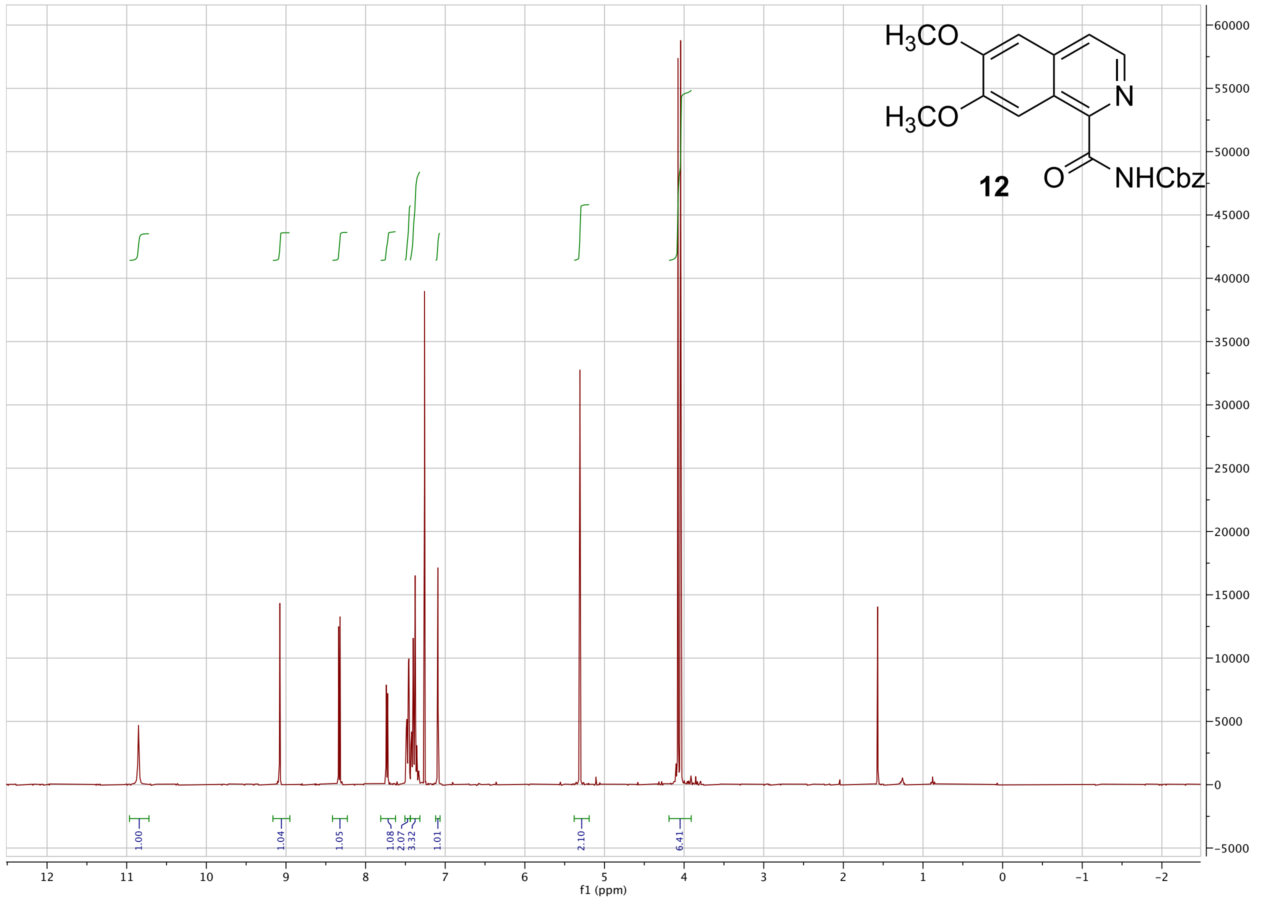

^{13}C-NMR (150 MHz)

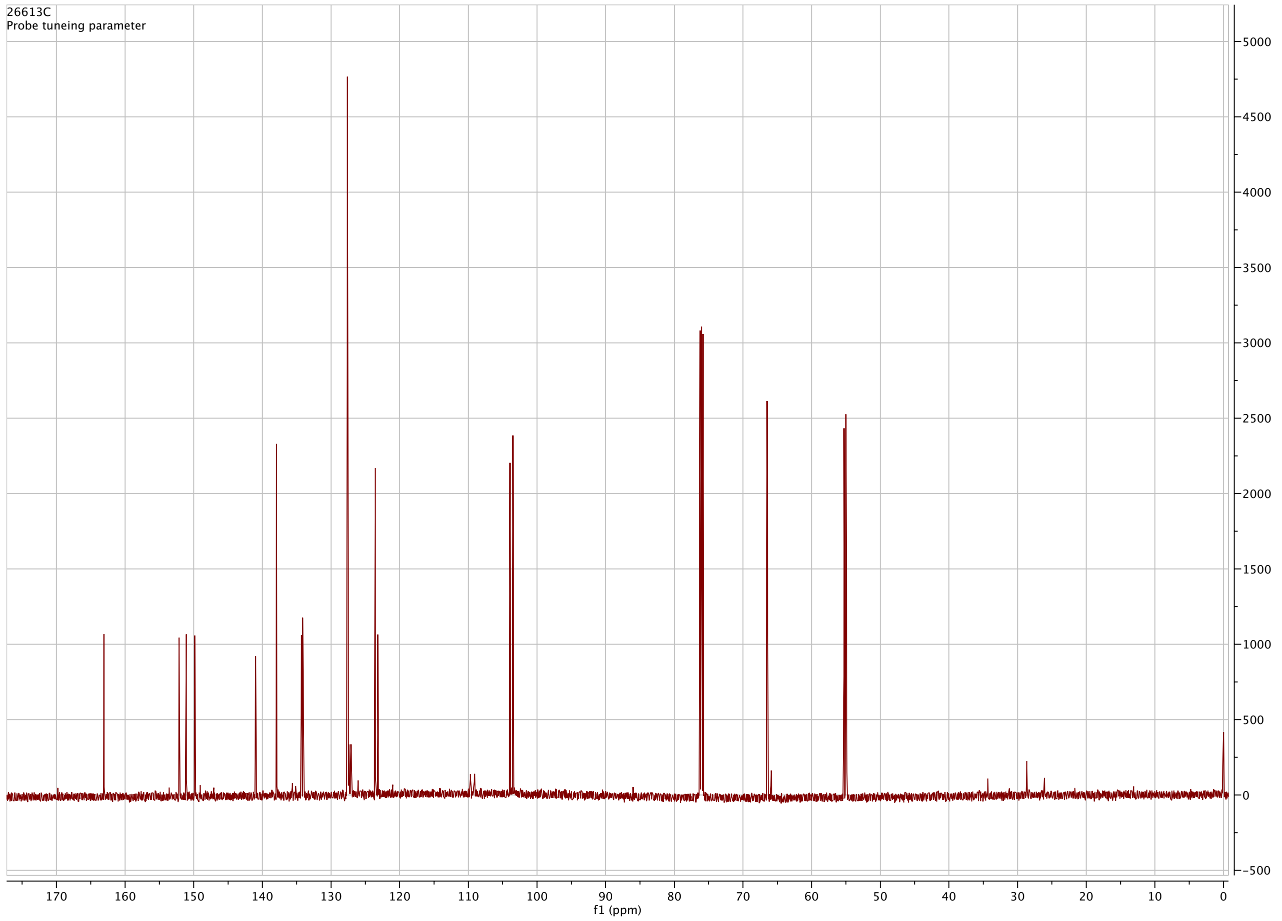

^{1}H-NMR (500 MHz)

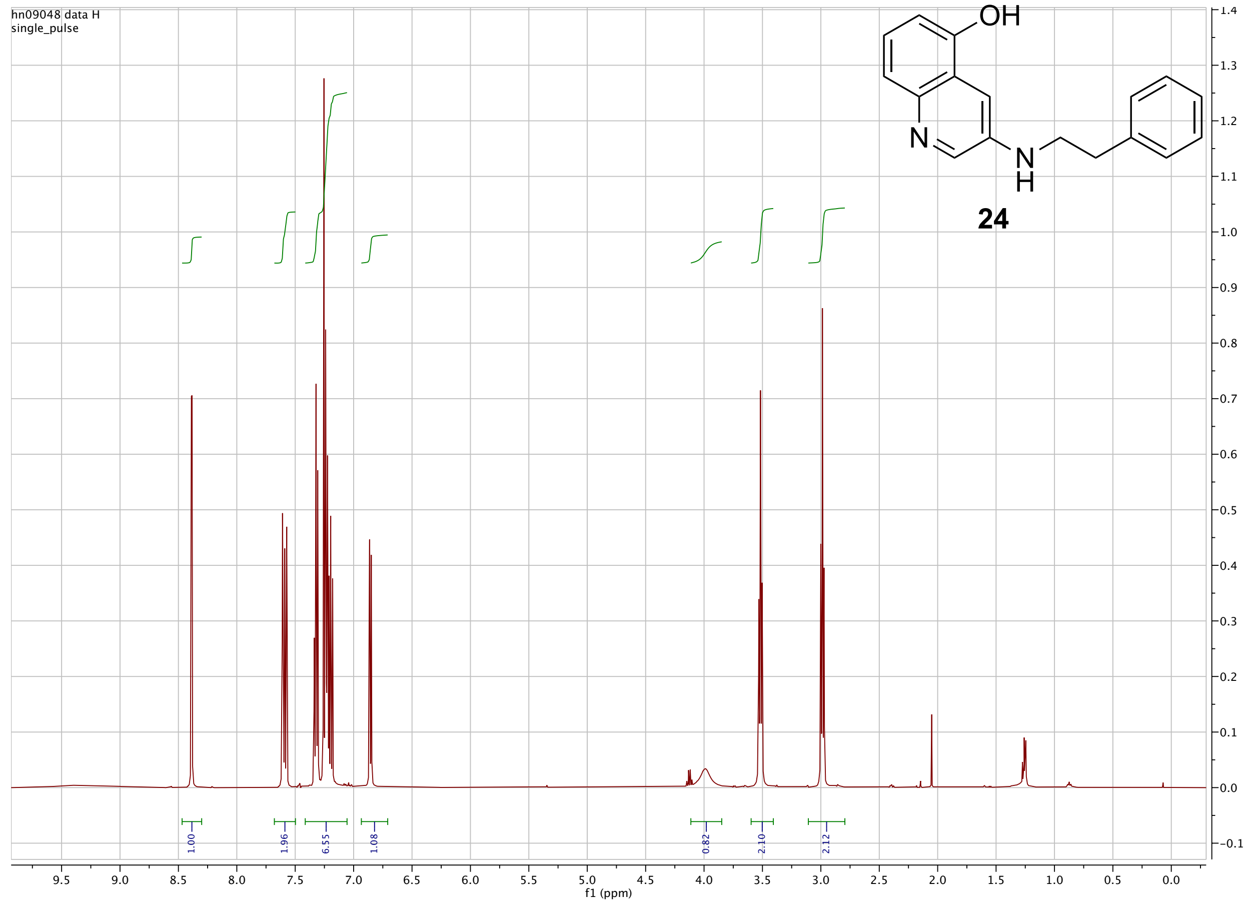

^{13}C-NMR (125 MHz)

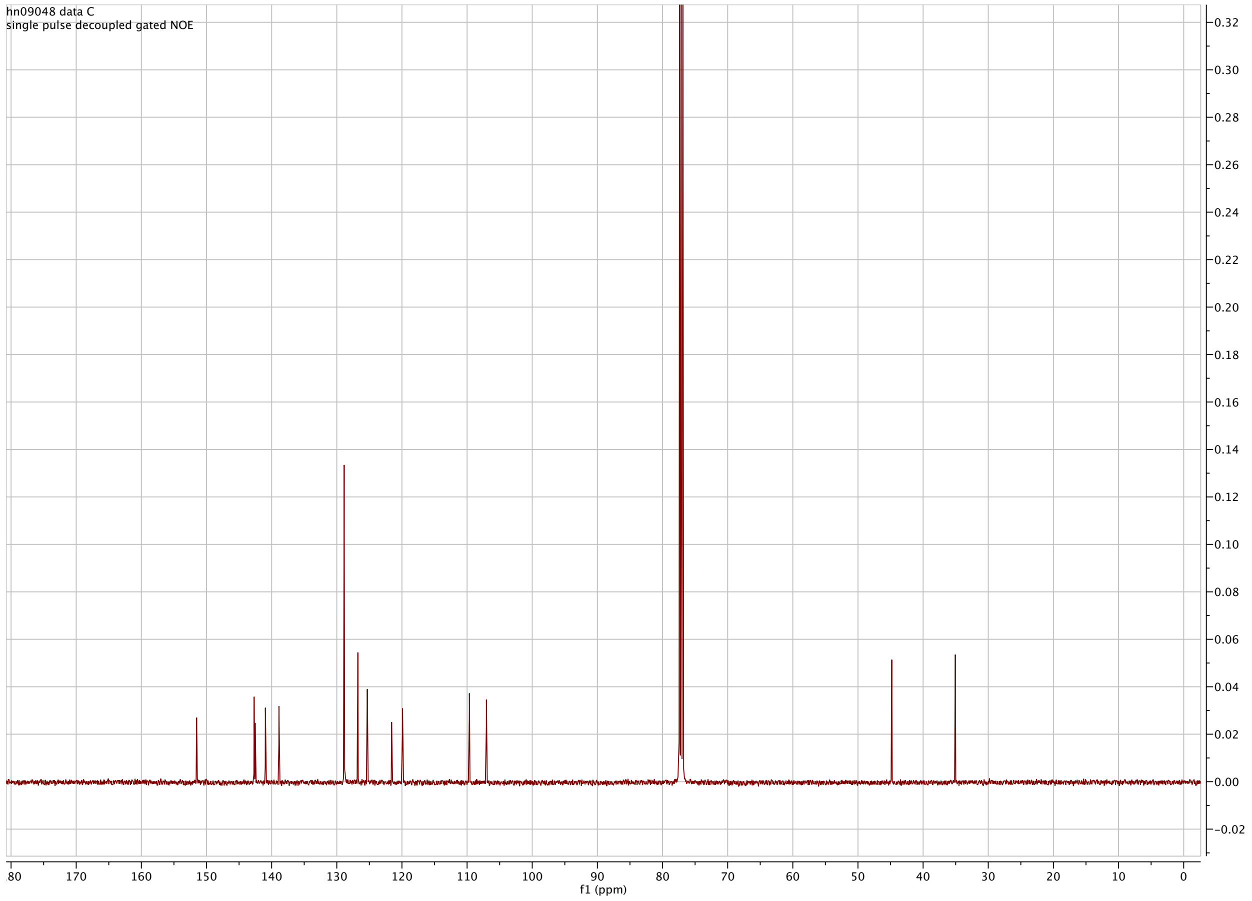

^{1}H-NMR (600 MHz)

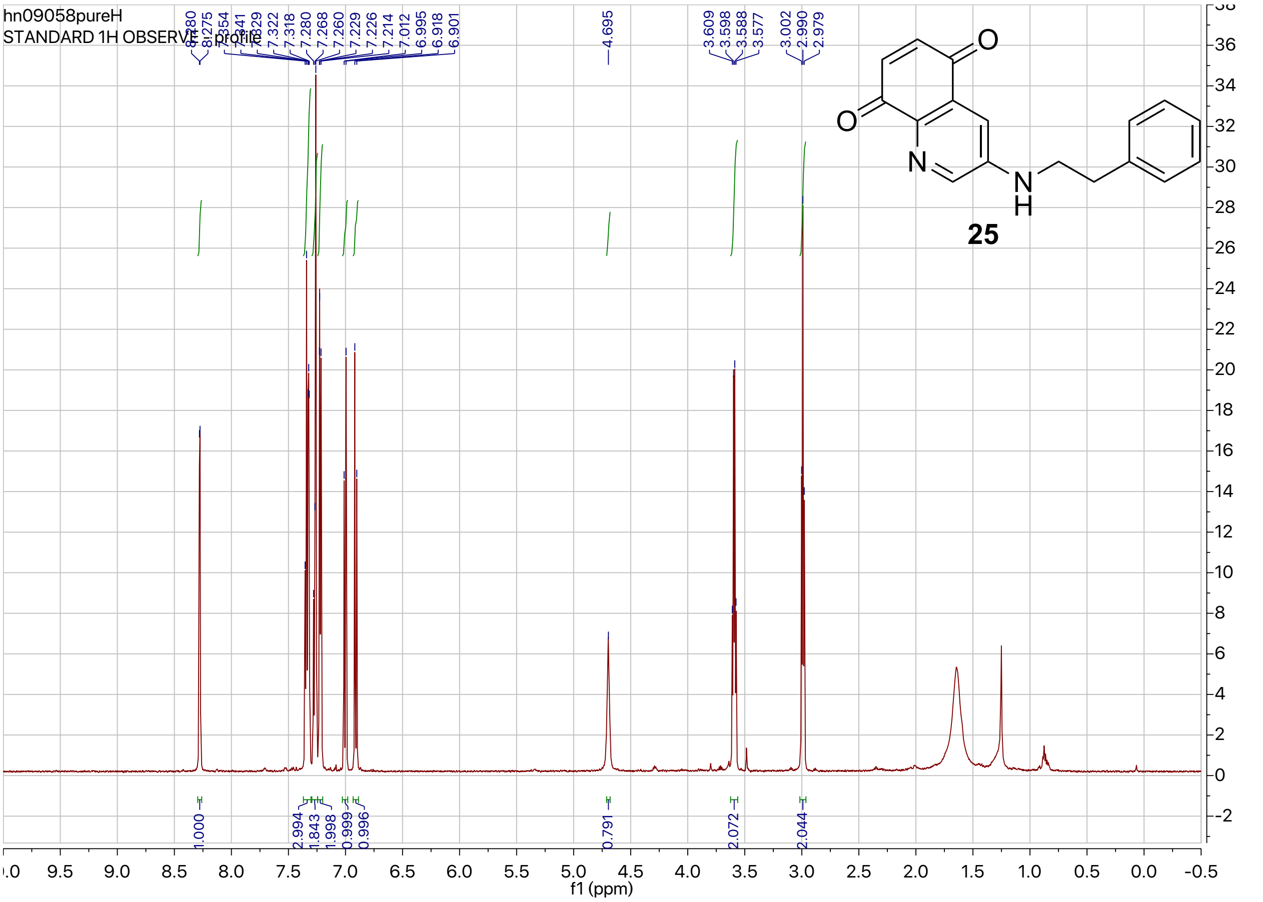

^{13}C-NMR (150 MHz)

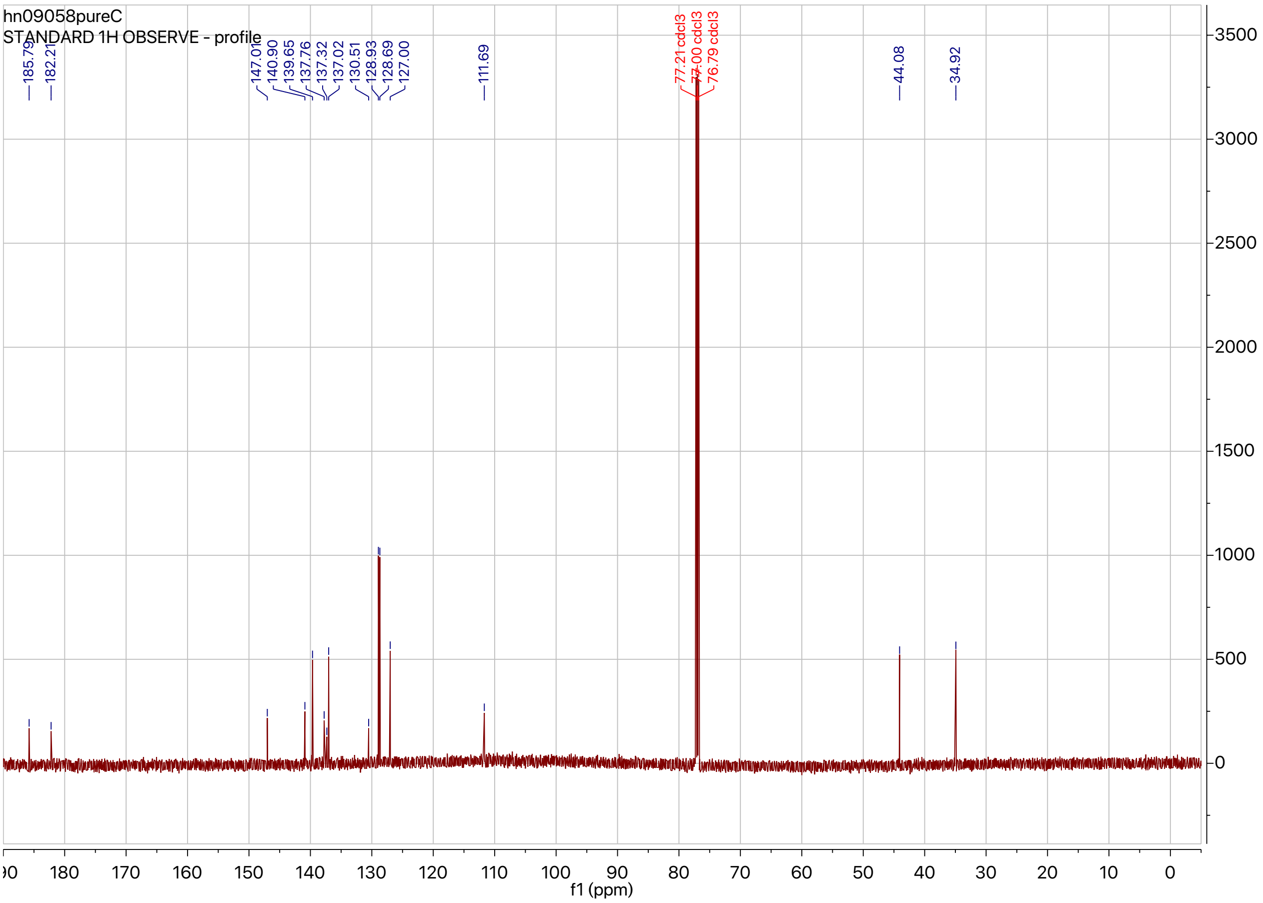

^{1}H-NMR (300 MHz)

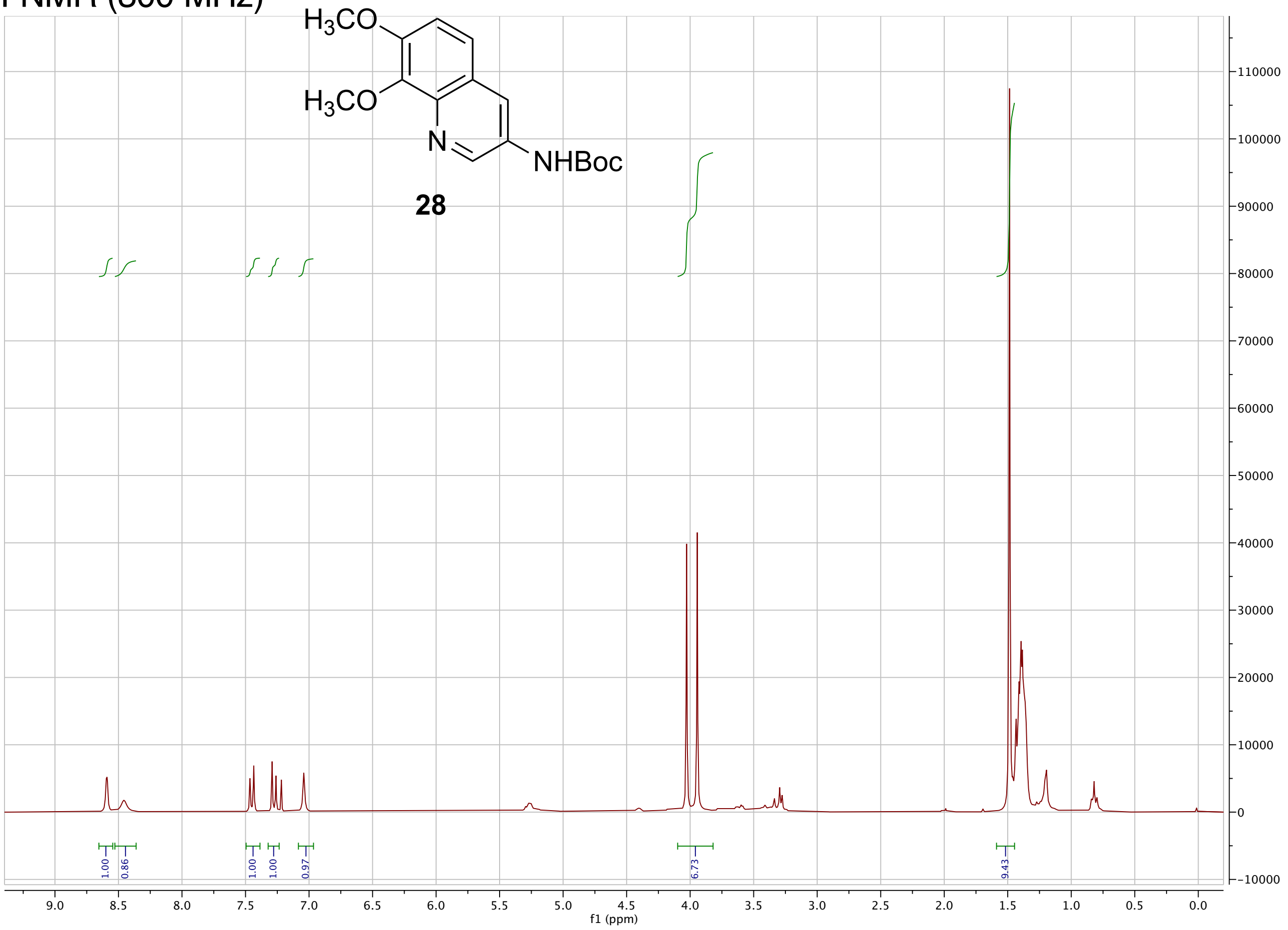

^{1}H-NMR (500 MHz)

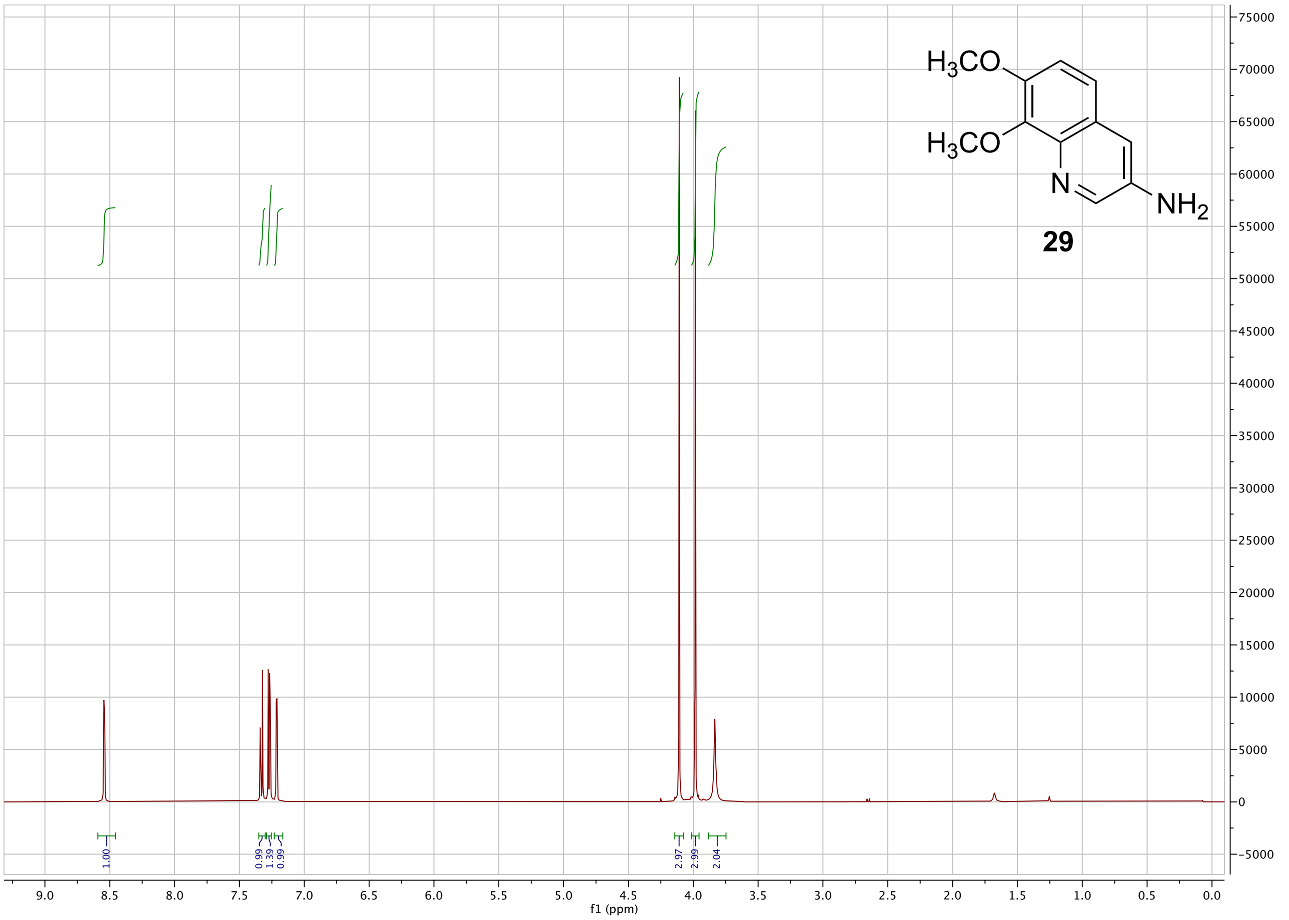

^{1}H-NMR (500 MHz)

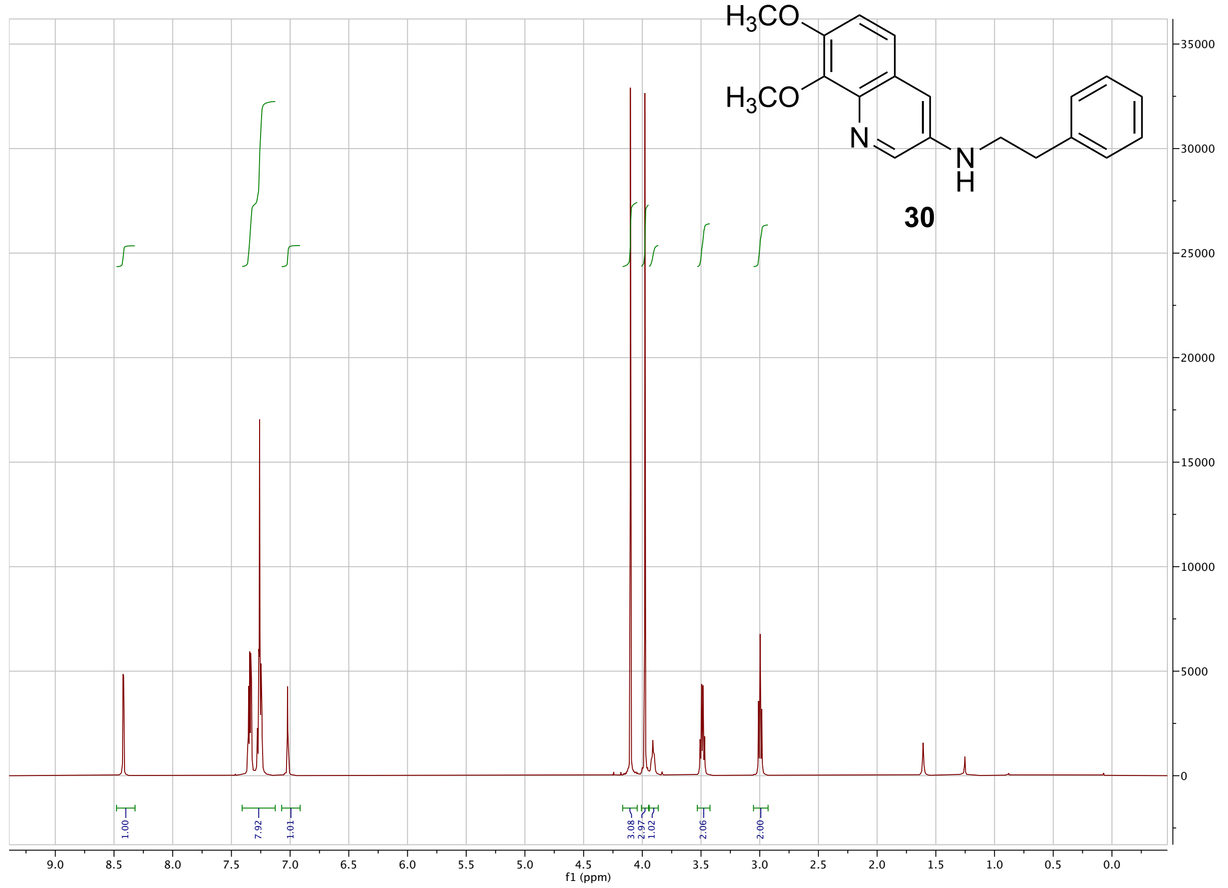

^{13}C-NMR (150 MHz)

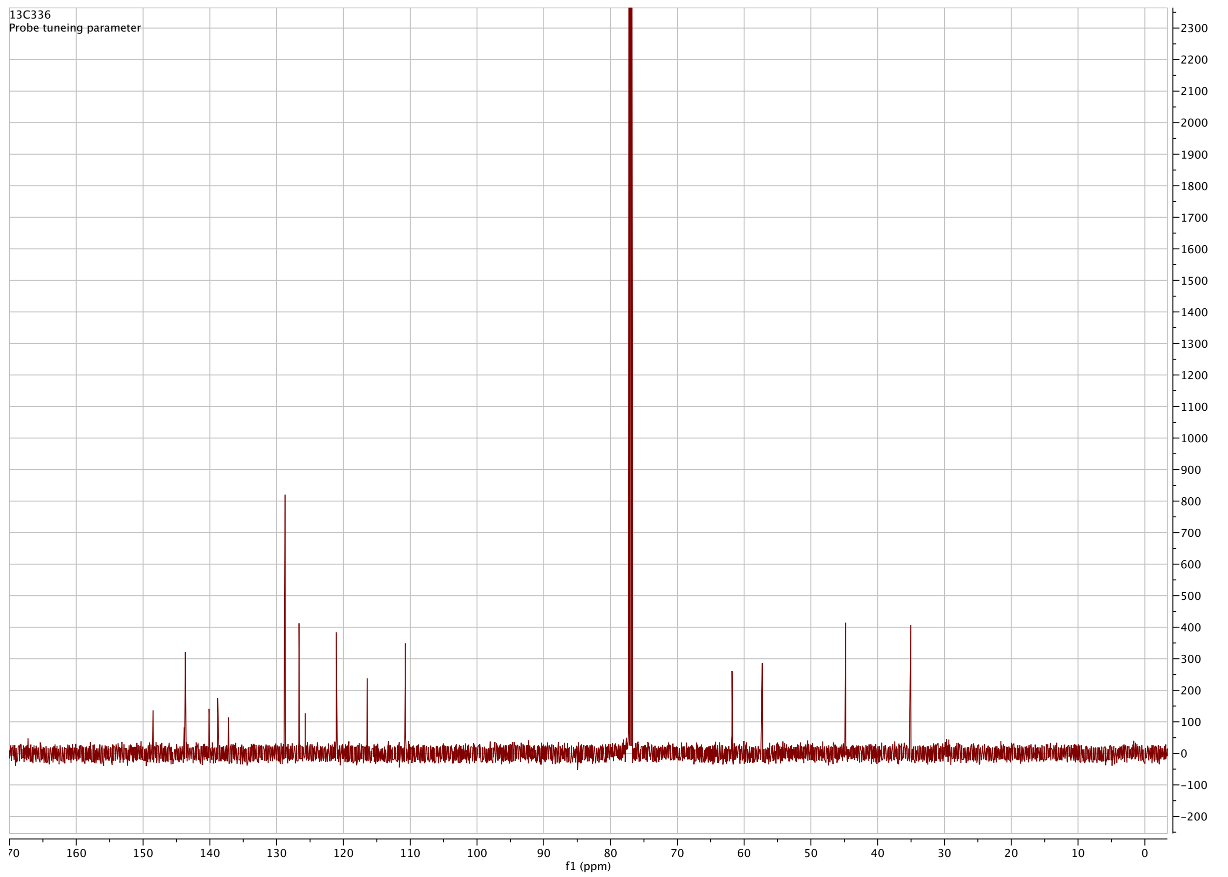

^{1}H-NMR (500 MHz)

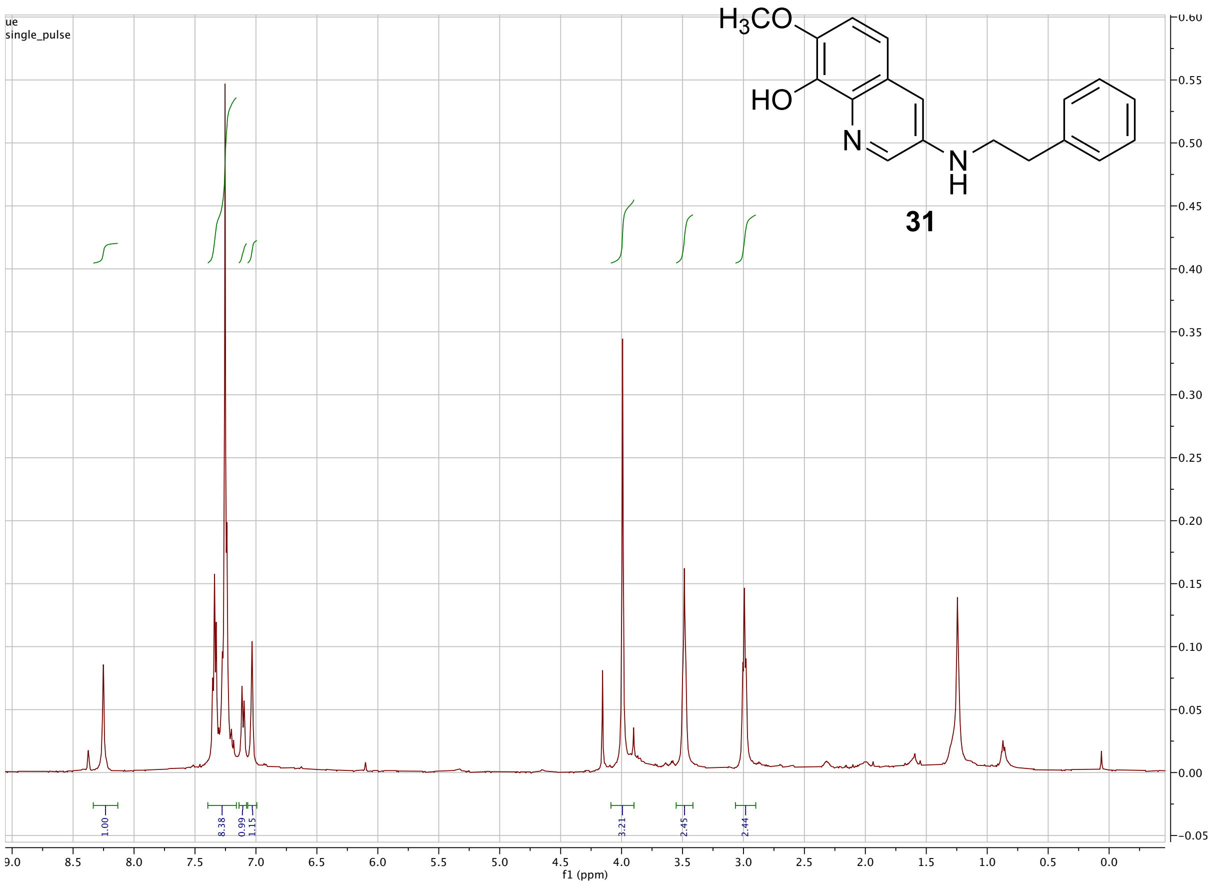

31

^{13}C-NMR (125 MHz)

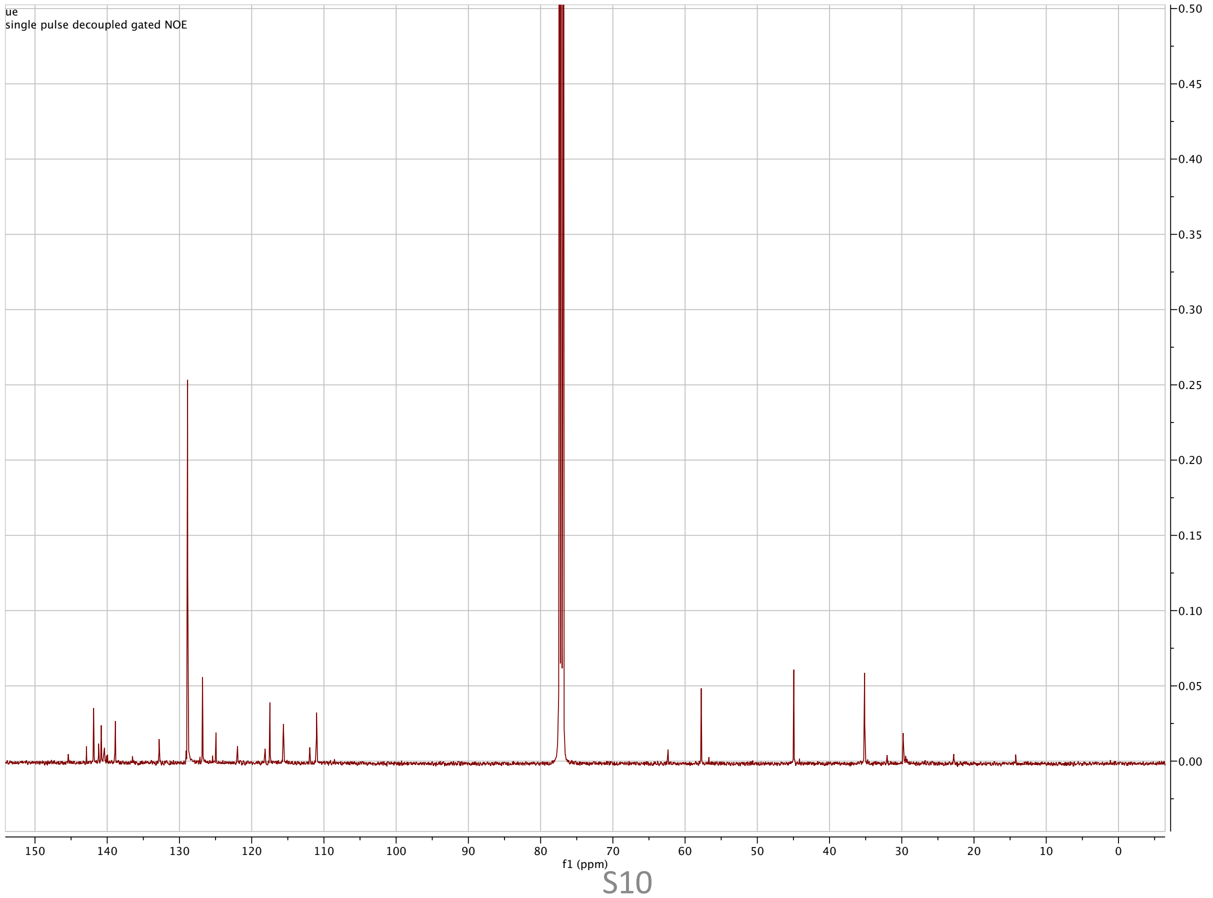

^{1}H-NMR (600 MHz)

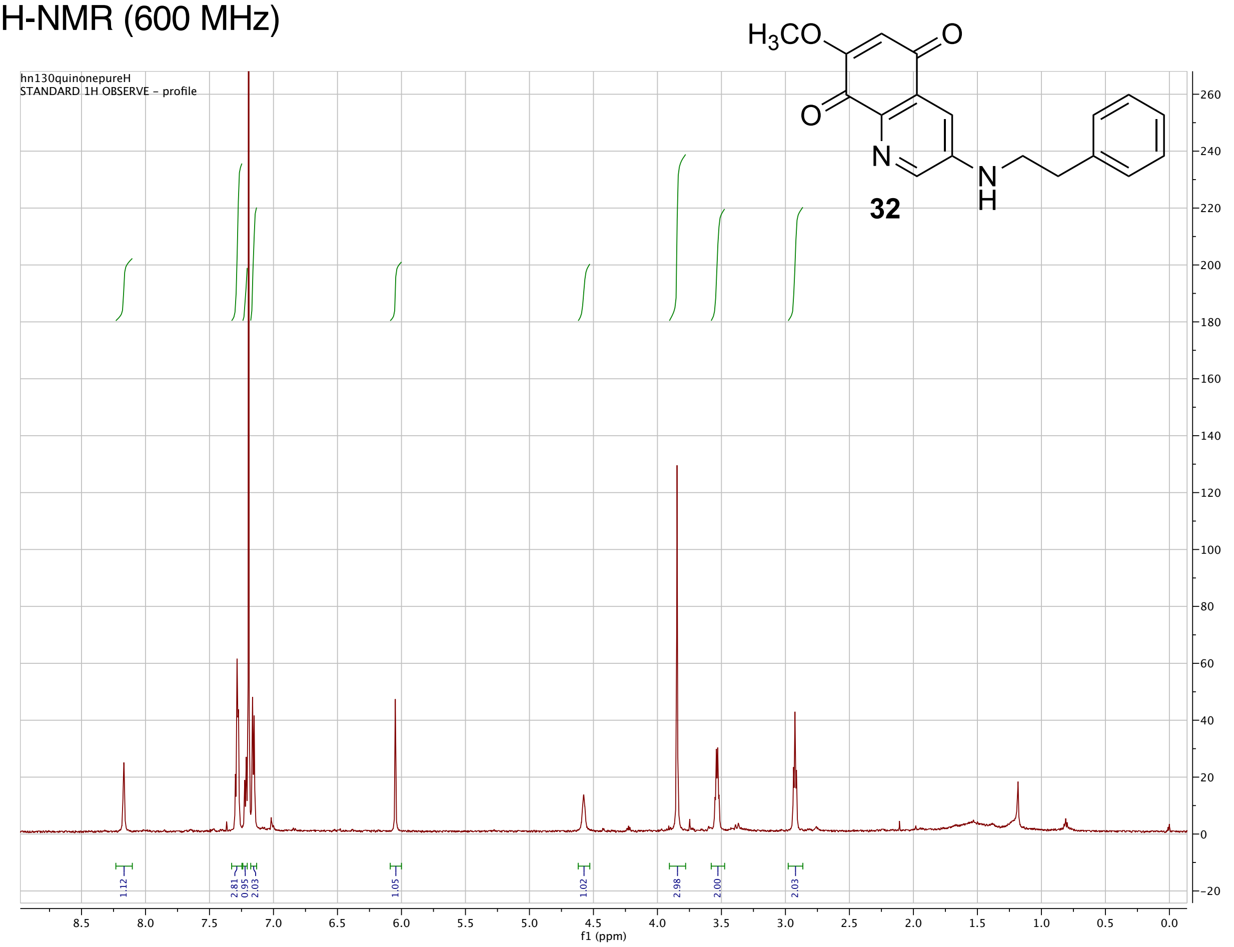

^{13}C-NMR (150 MHz)

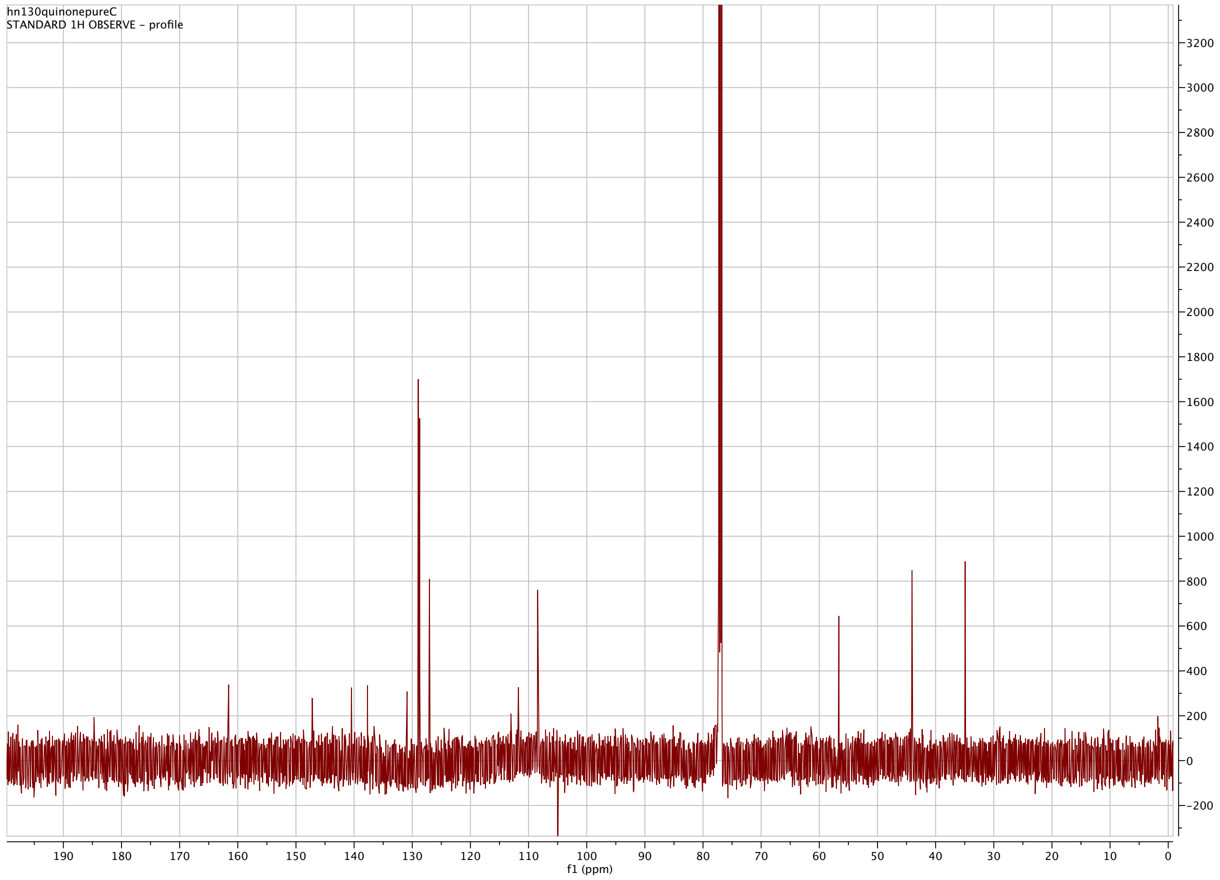

^{1}H-NMR (600 MHz)

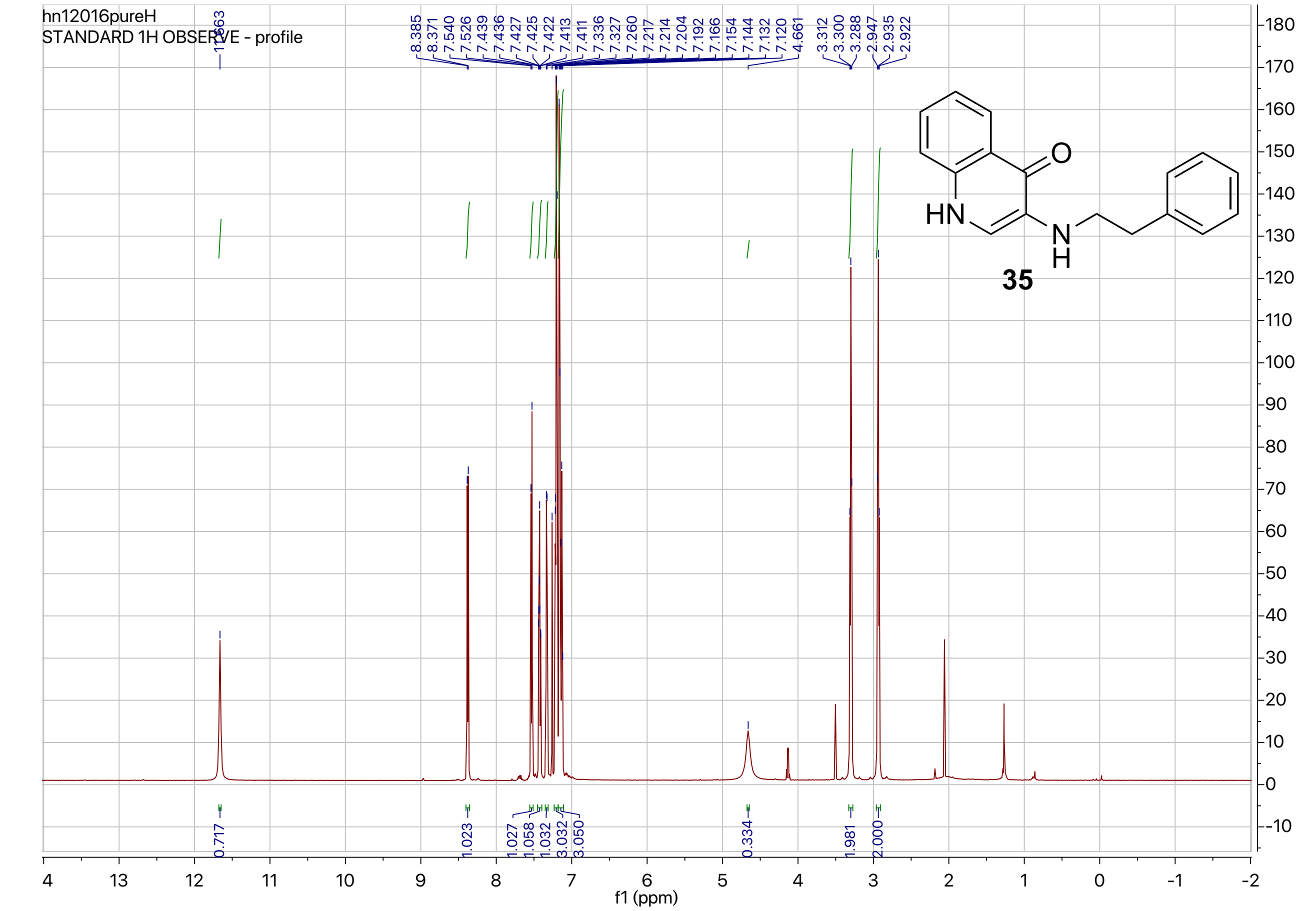

^{13}C-NMR (150 MHz)

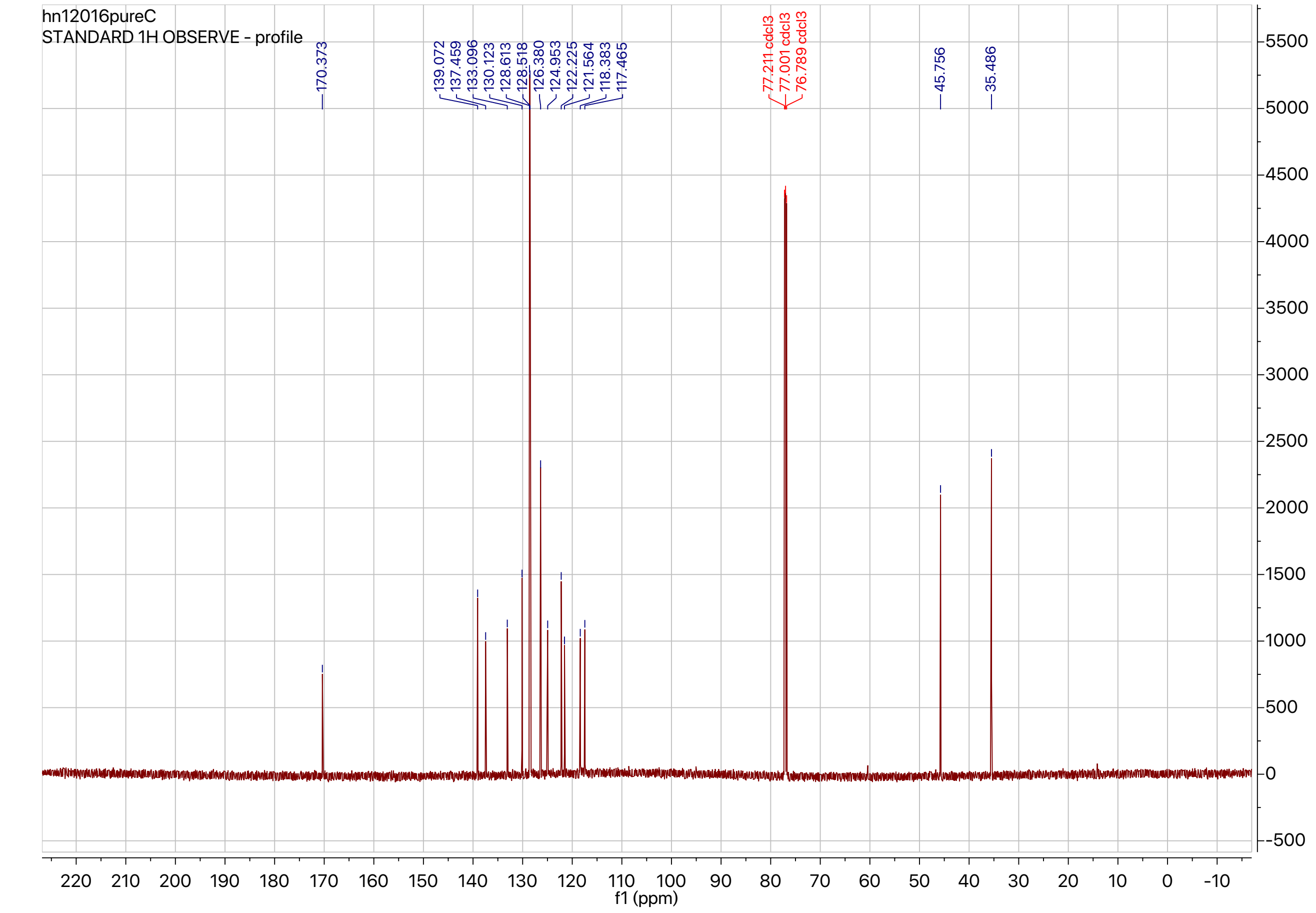

^{1}H-NMR (600 MHz)

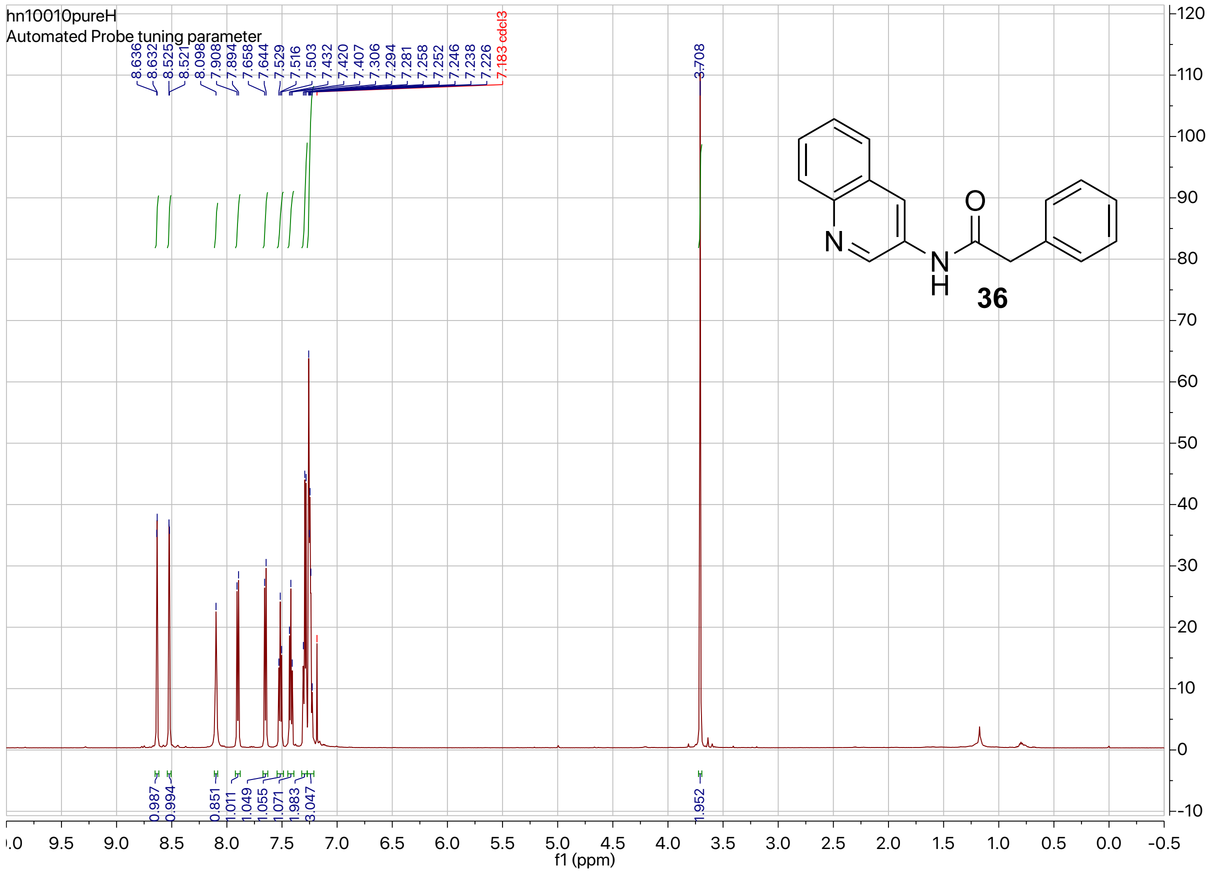

^{13}C-NMR (150 MHz)

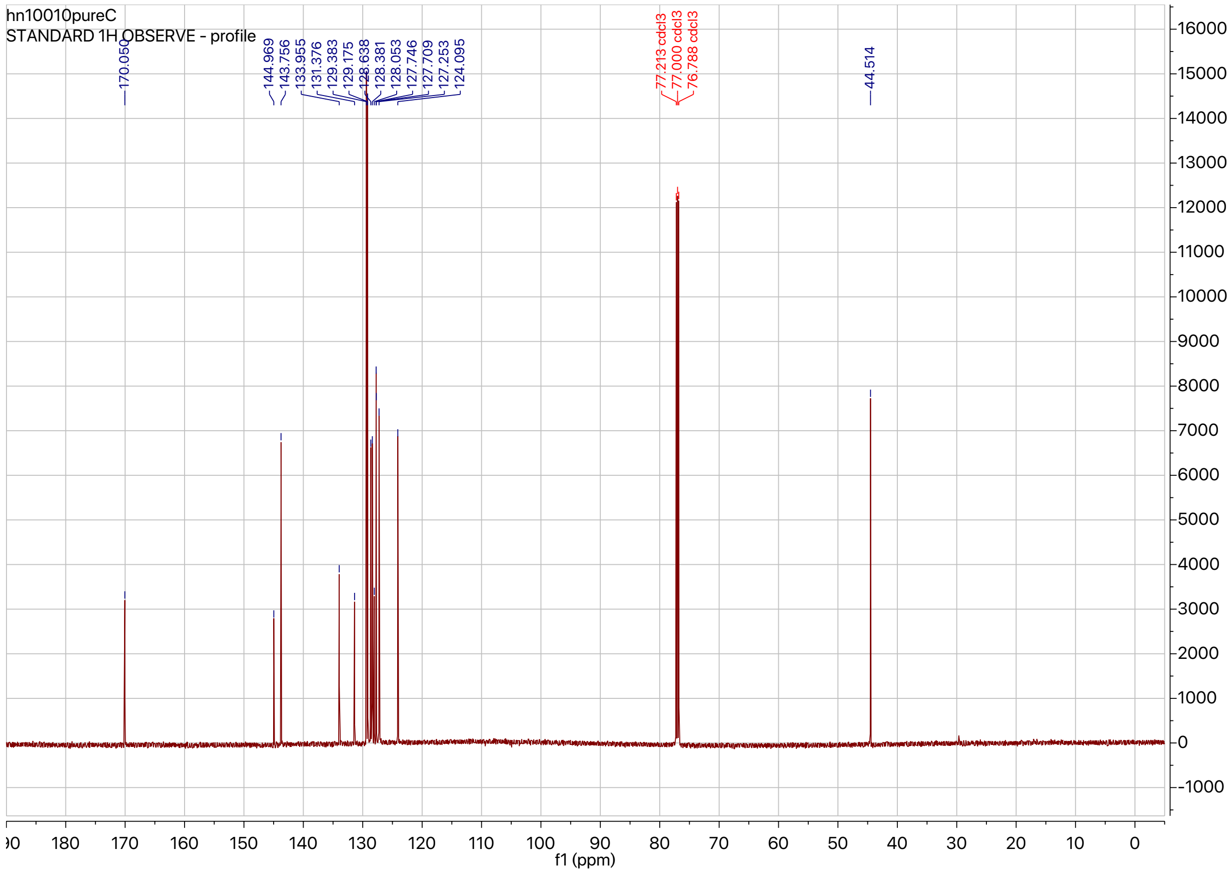

^{1}H-NMR (600 MHz)

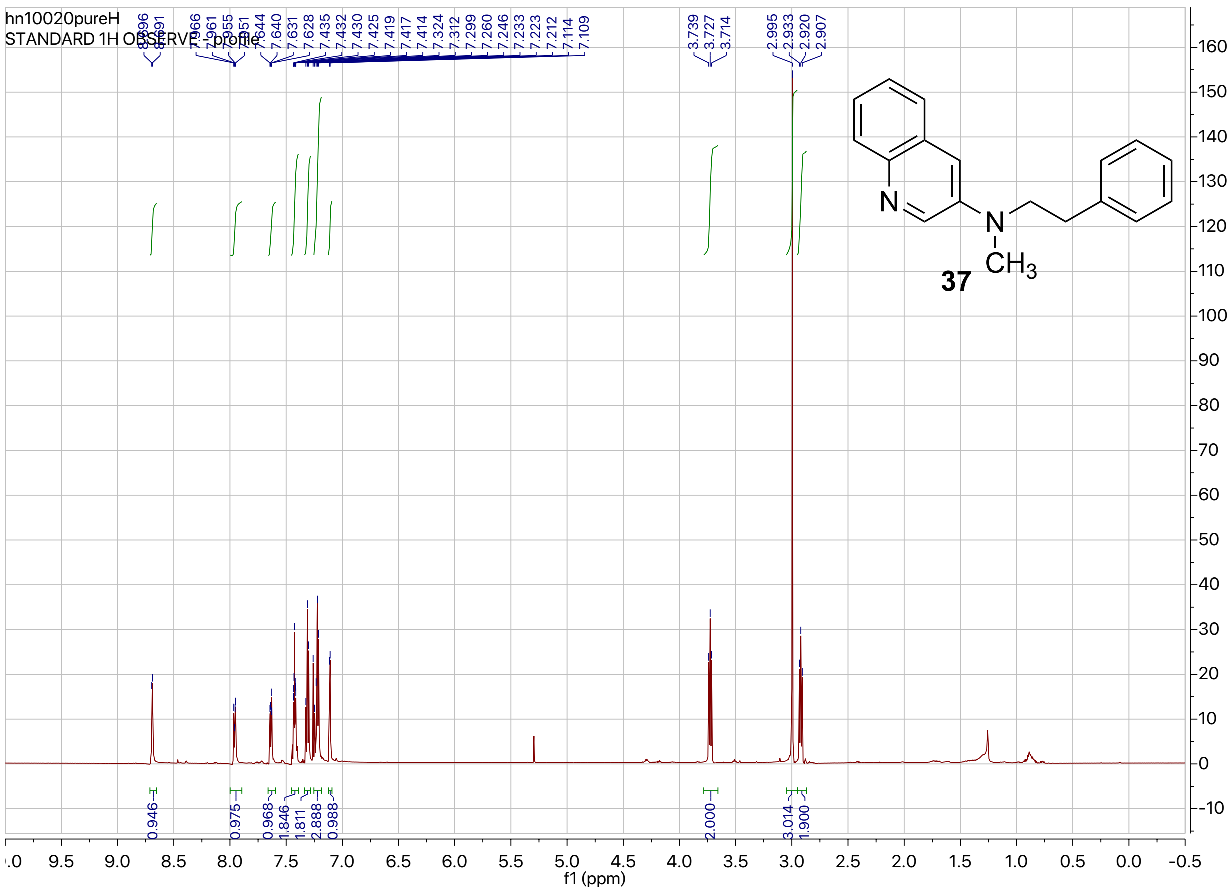

^{13}C-NMR (150 MHz)

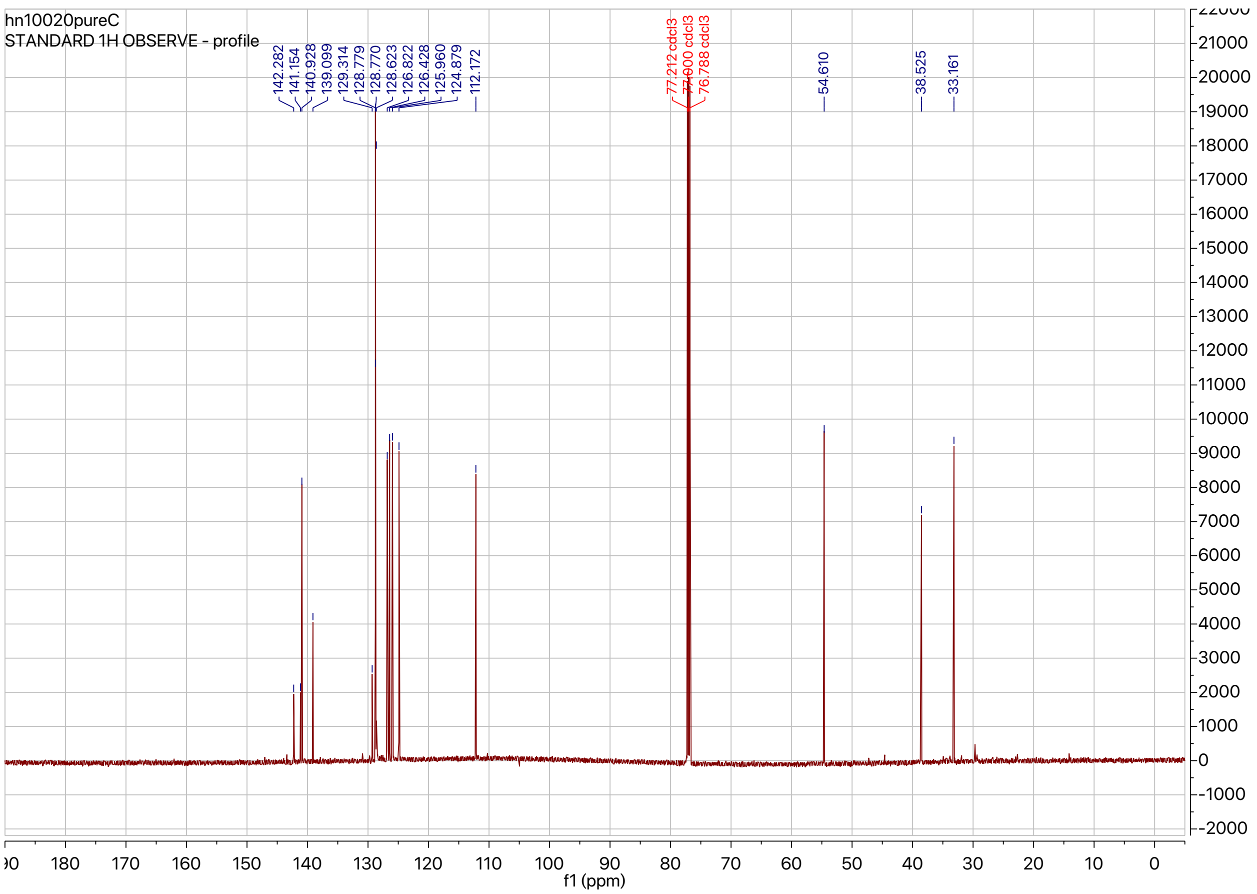

^{1}H-NMR (500 MHz)

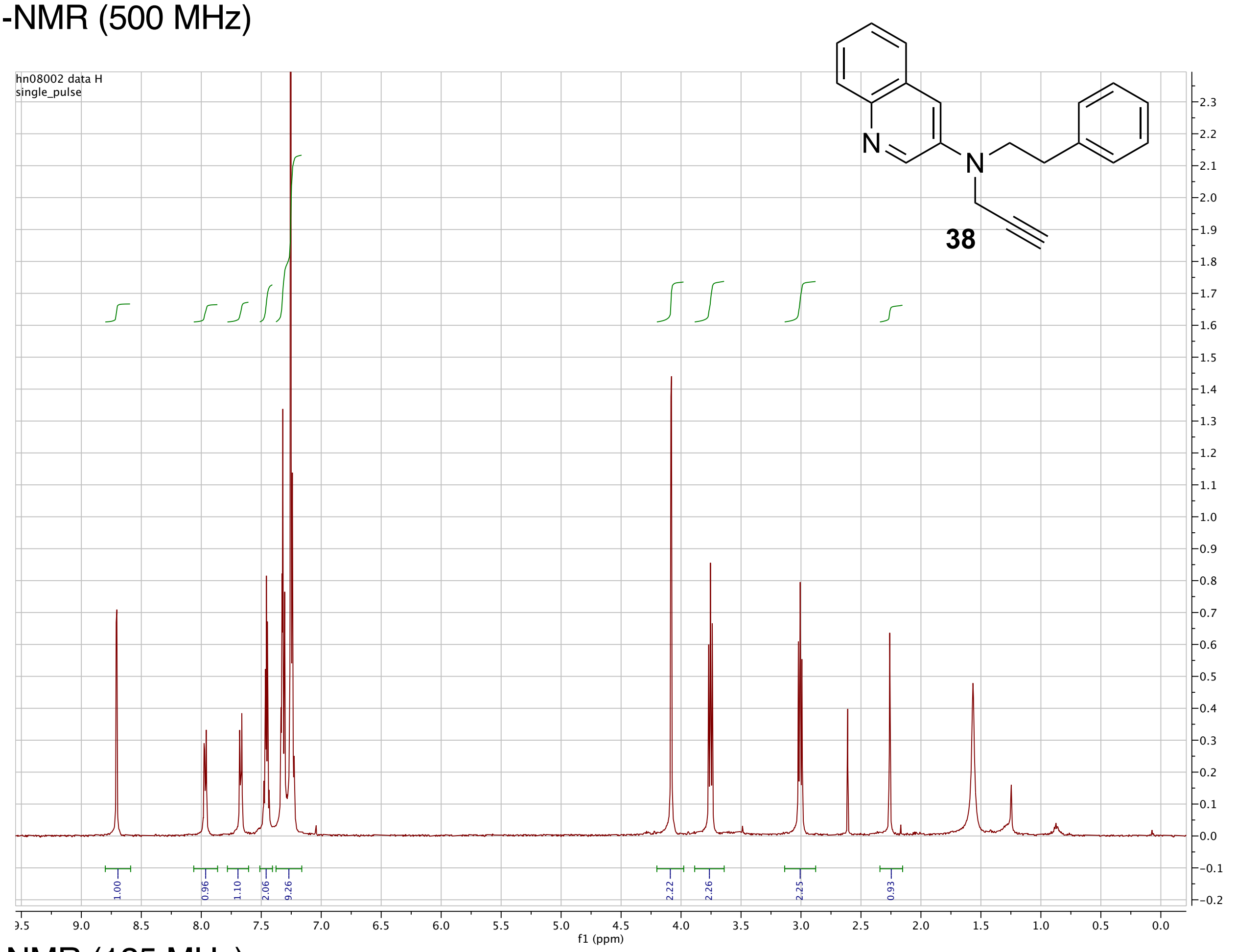

^{13}C-NMR (125 MHz)

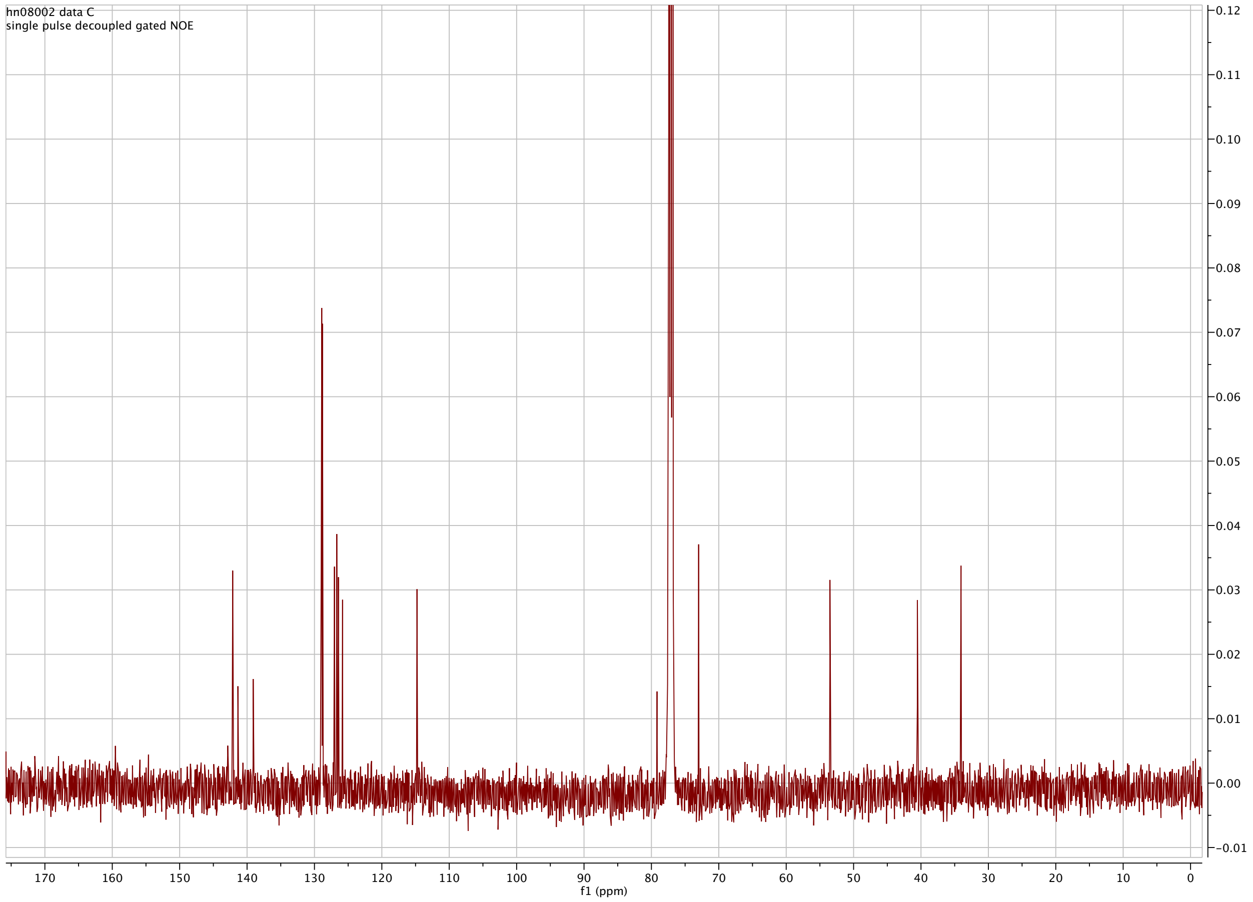

^{1}H-NMR (600 MHz)

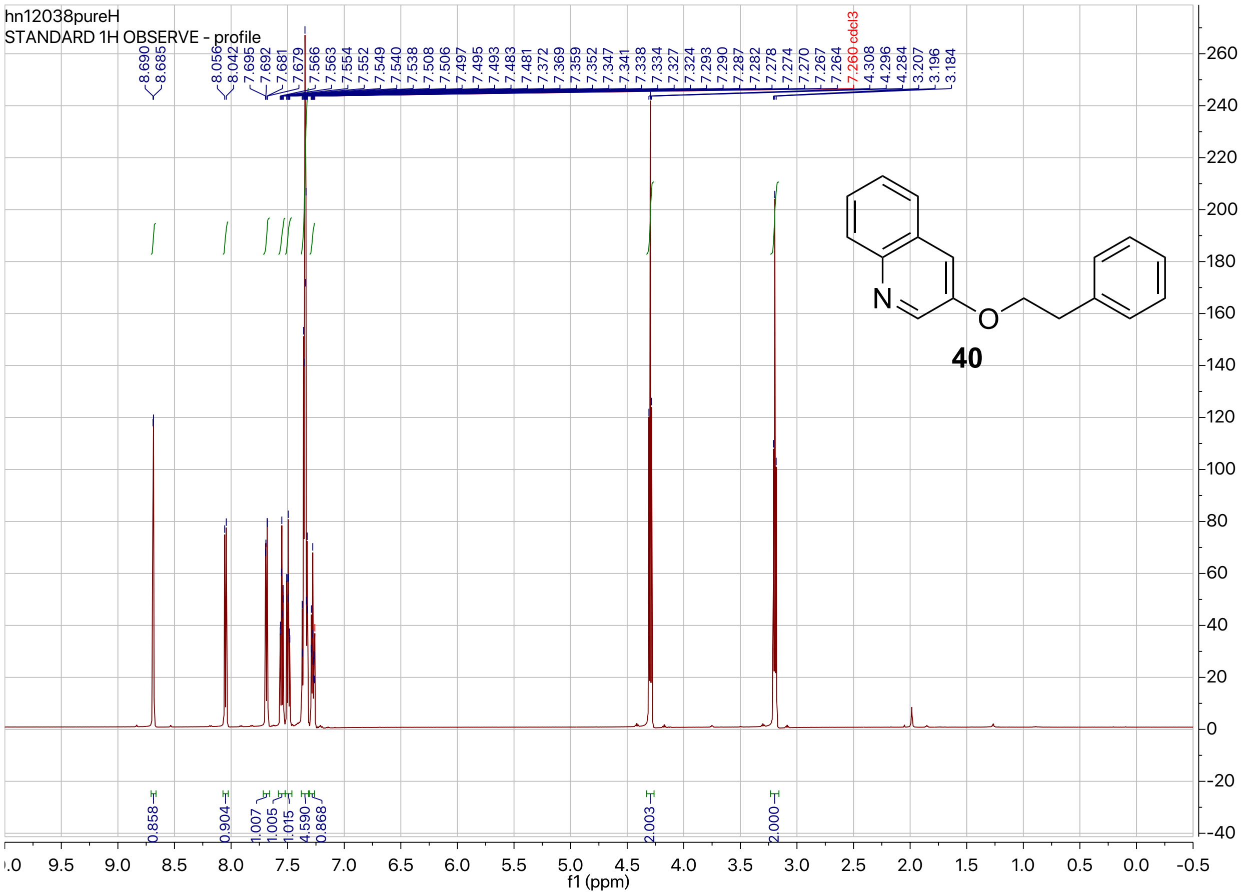

^{13}C-NMR (150 MHz)

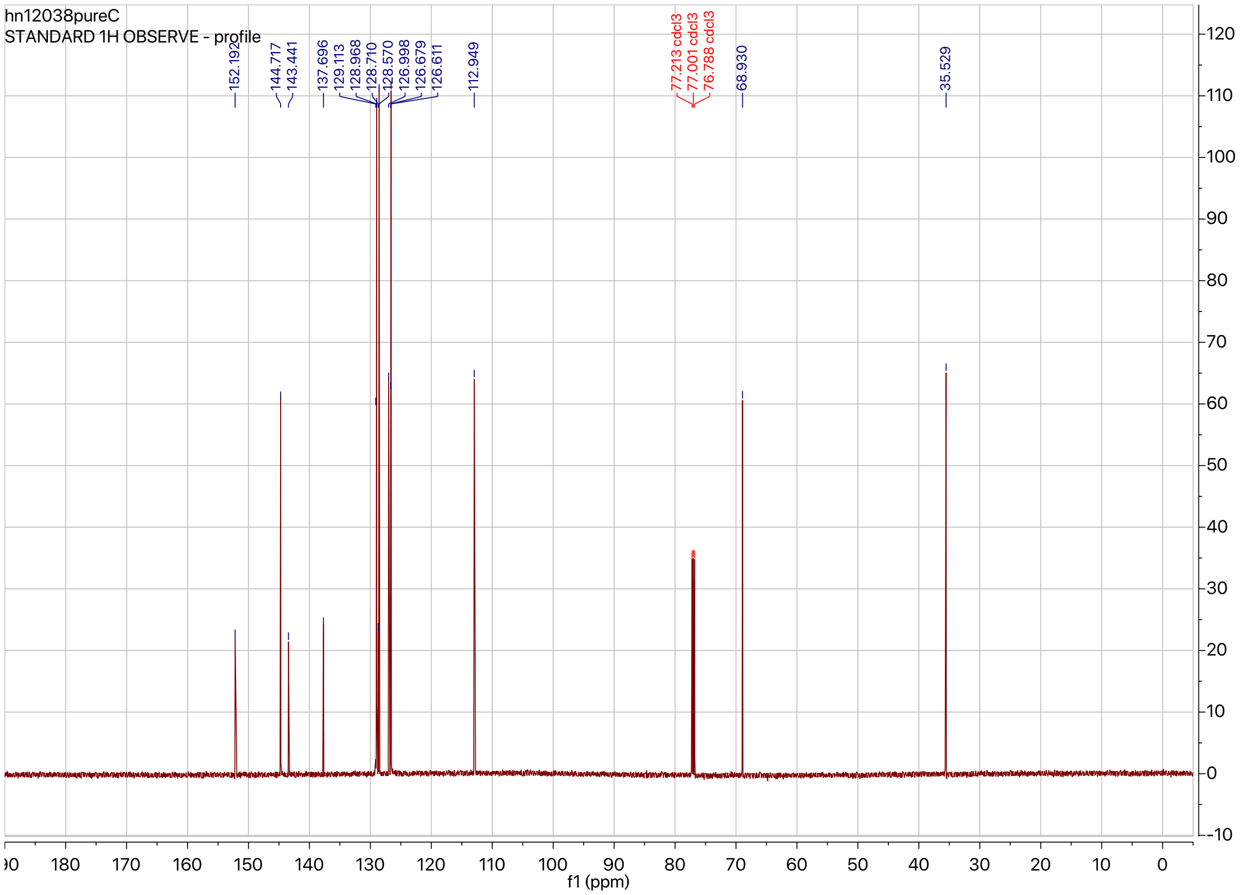

^{1}H-NMR (500 MHz)

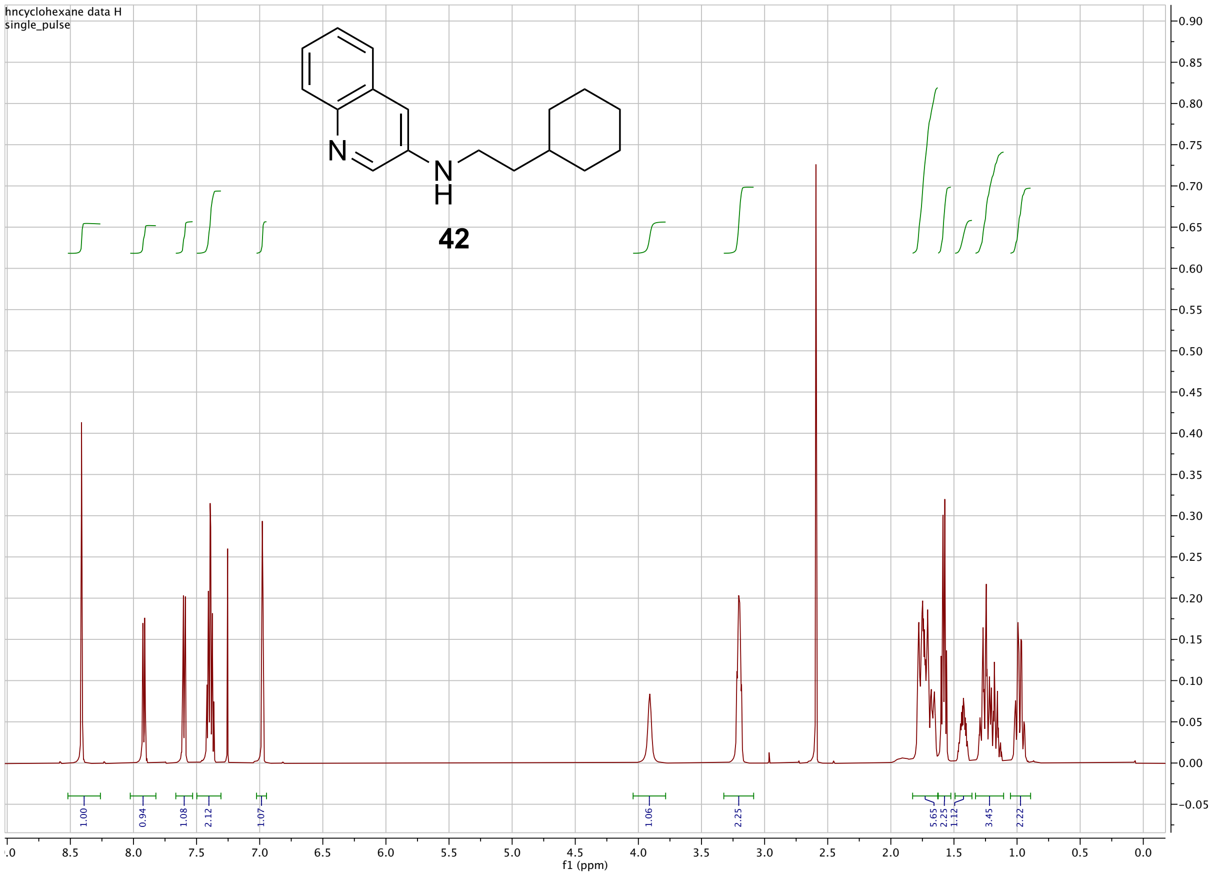

^{13}C-NMR (125 MHz)

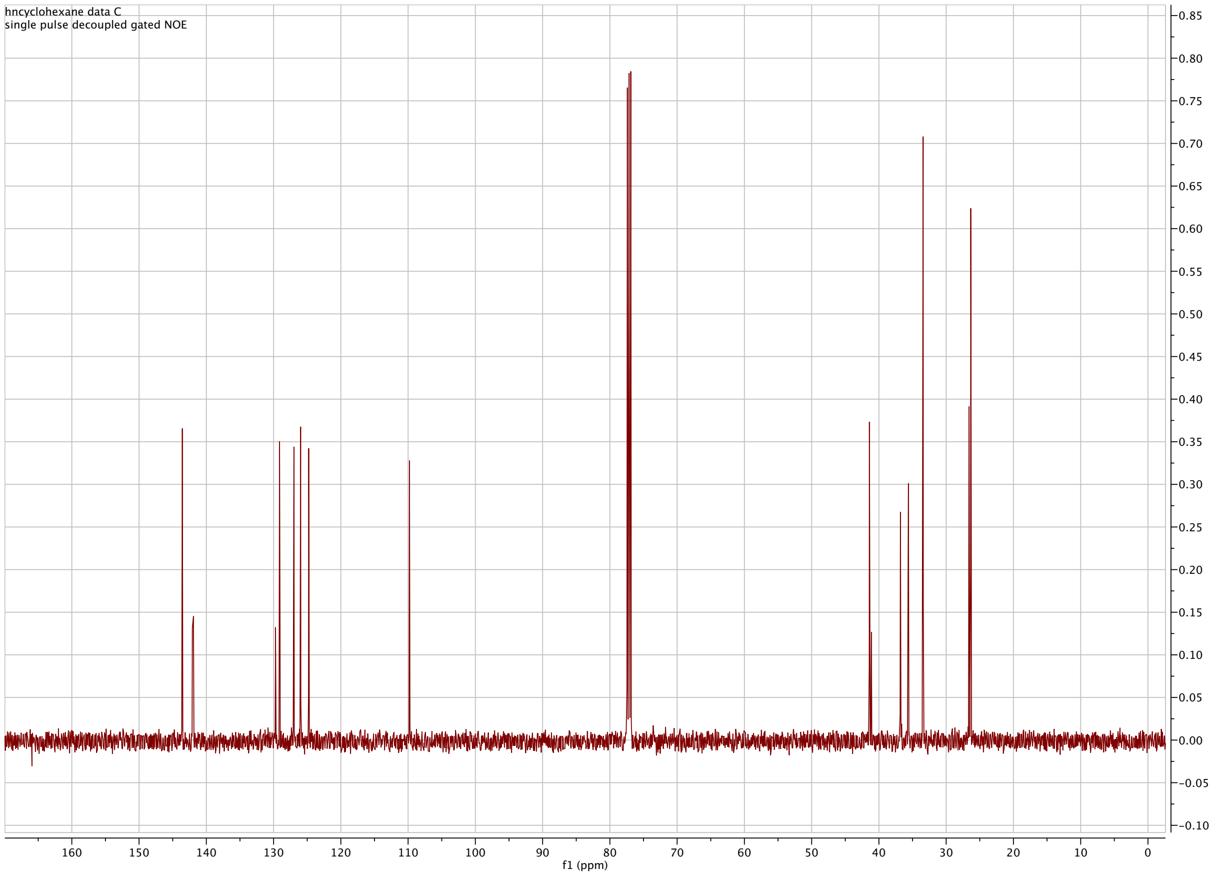

^{1}H-NMR (600 MHz)

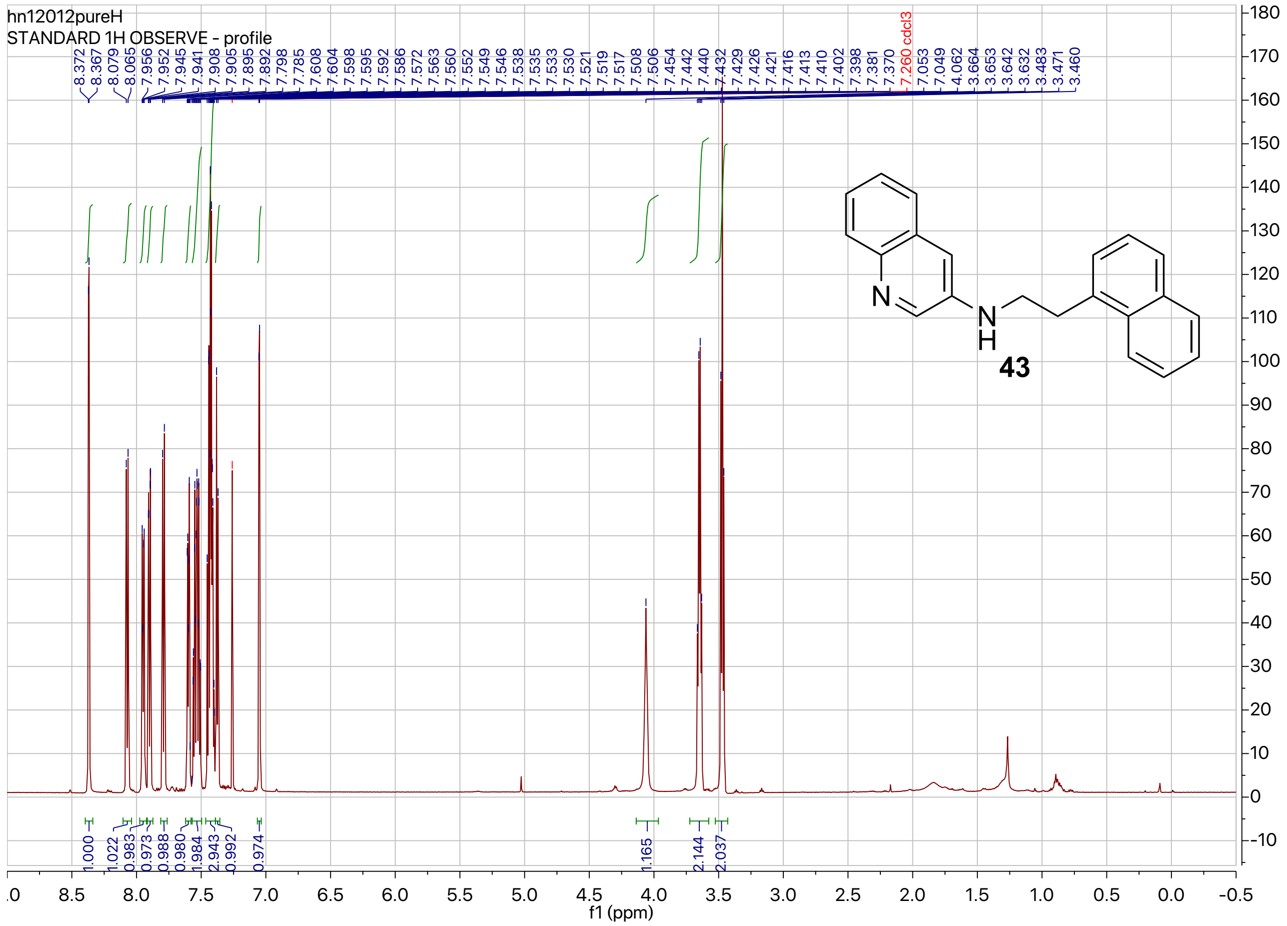

^{13}C-NMR (150 MHz)

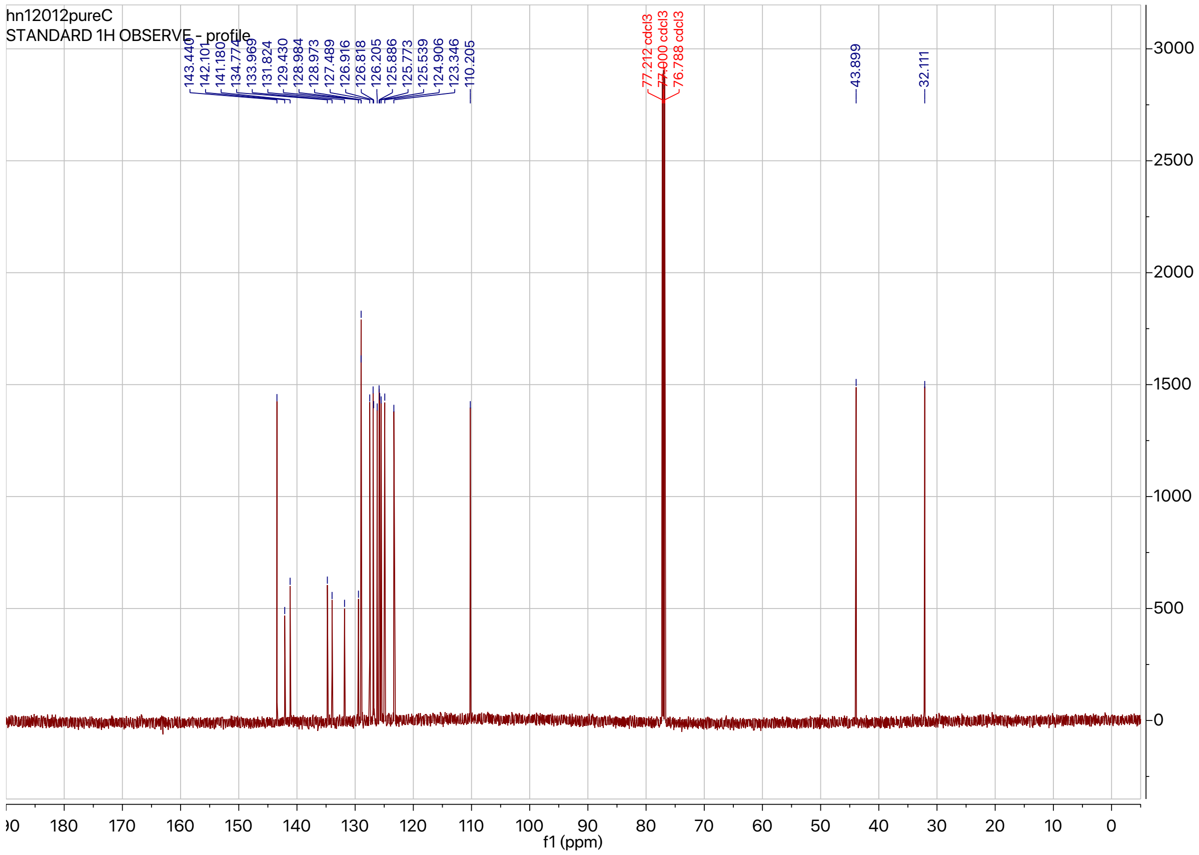

^{1}H-NMR (500 MHz)

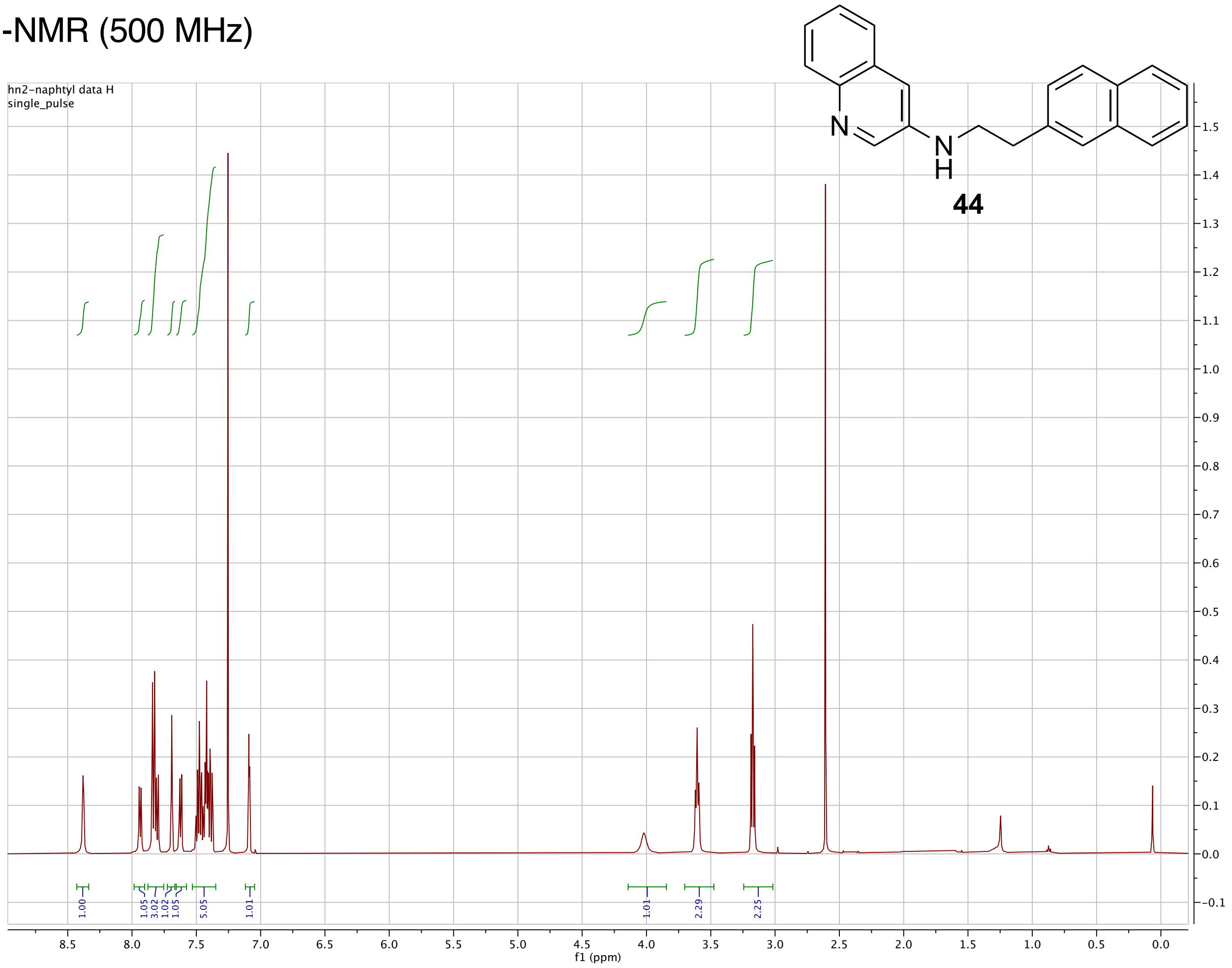

44

^{13}C-NMR (125 MHz)

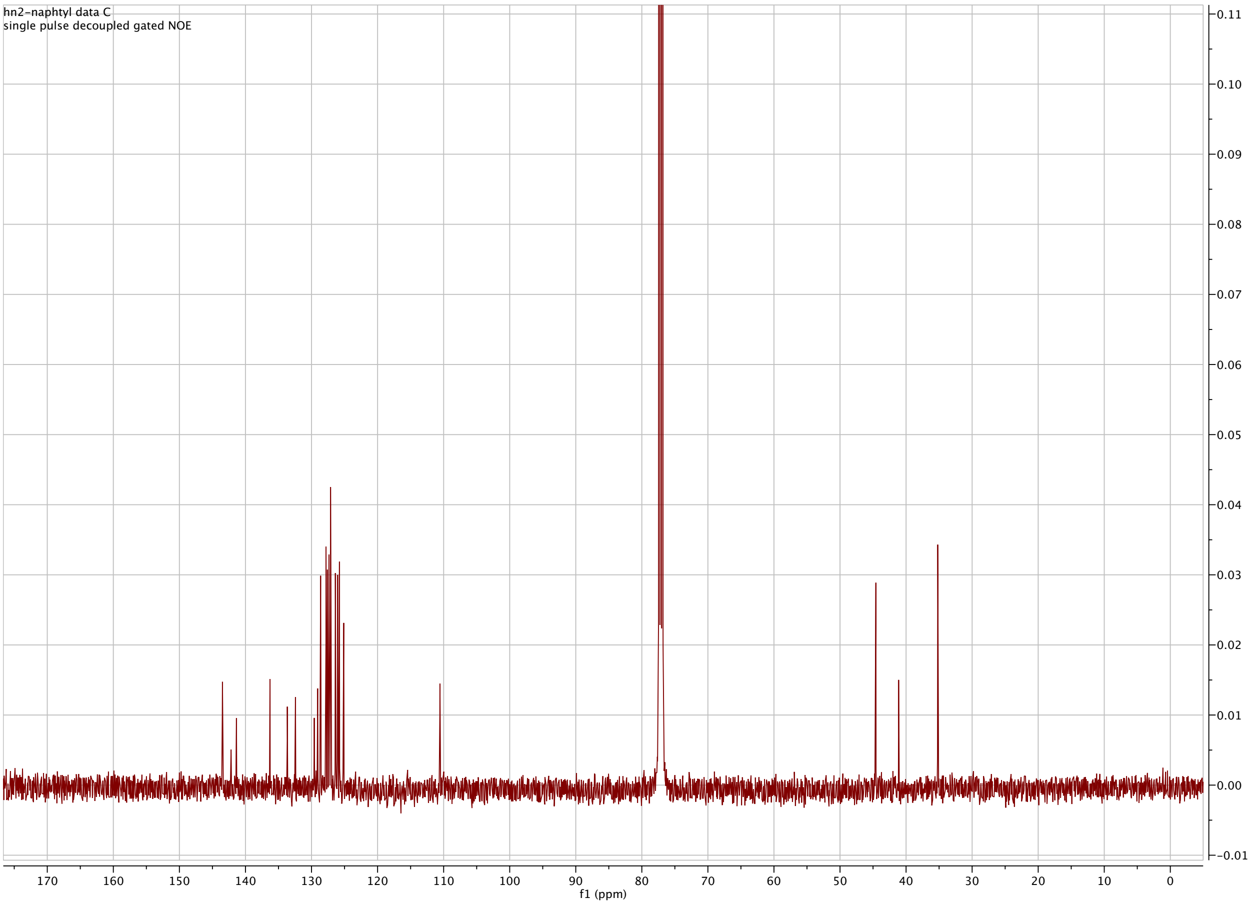

^{1}H-NMR (500 MHz)

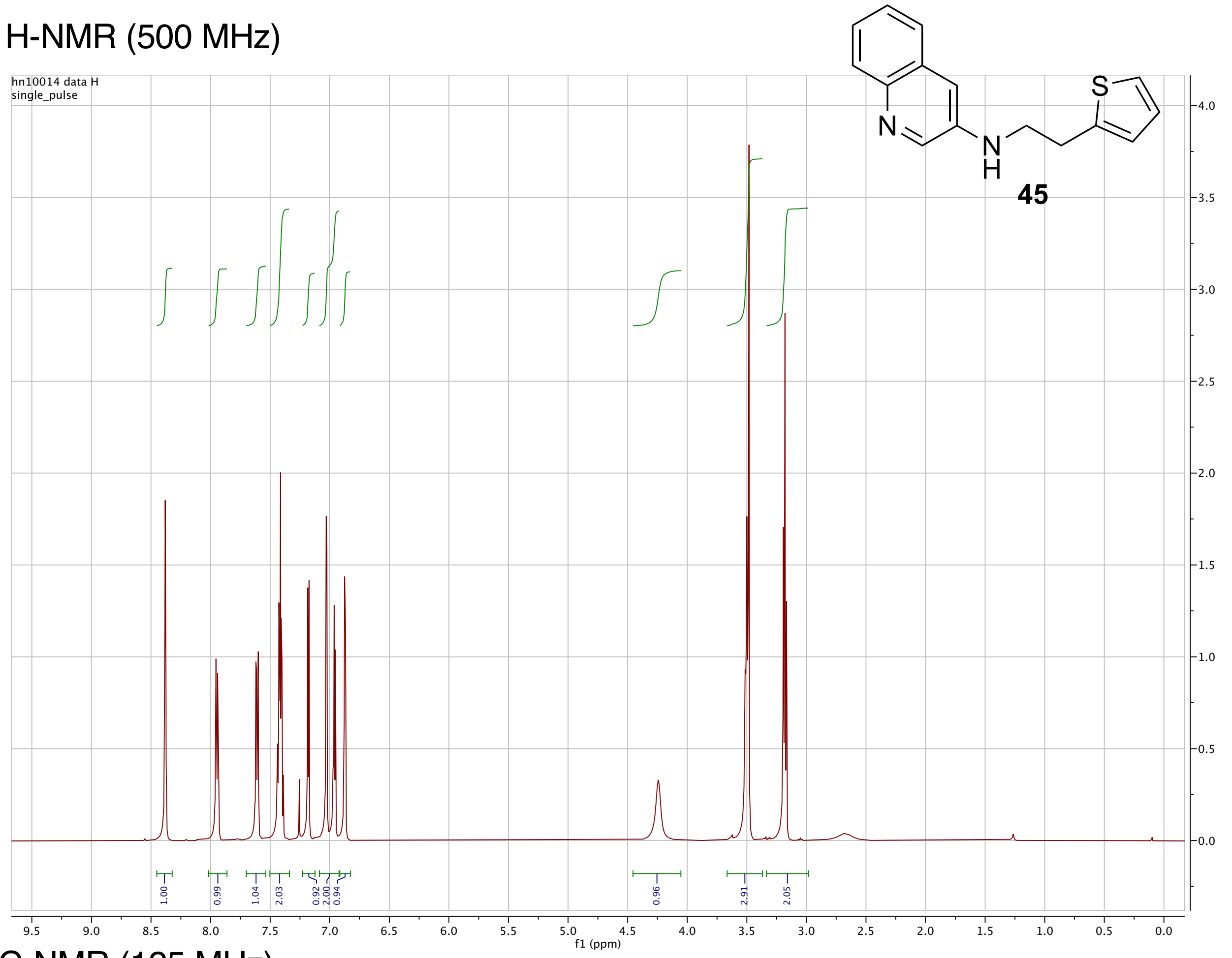

^{13}C-NMR (125 MHz)

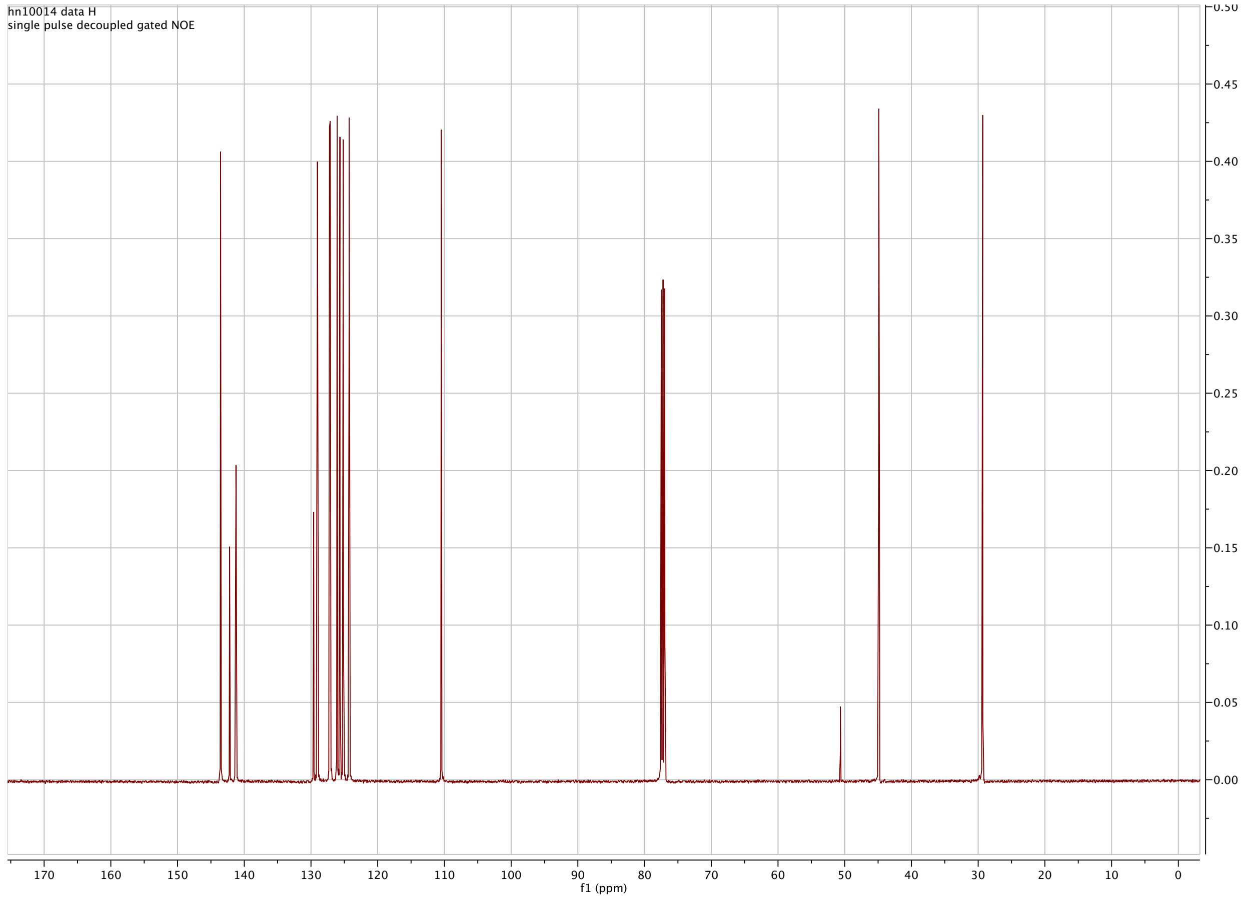

^{1}H-NMR (600 MHz)

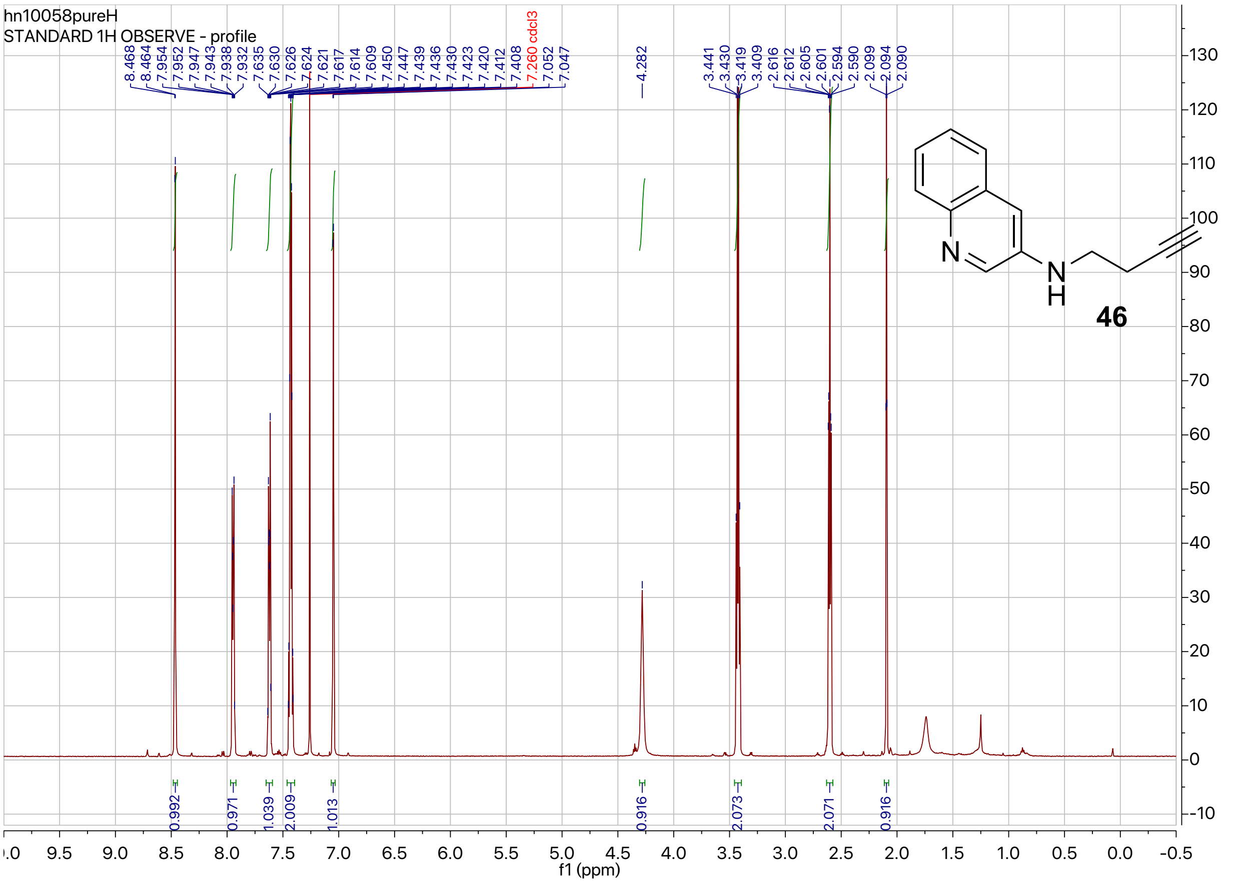

^{13}C-NMR (150 MHz)

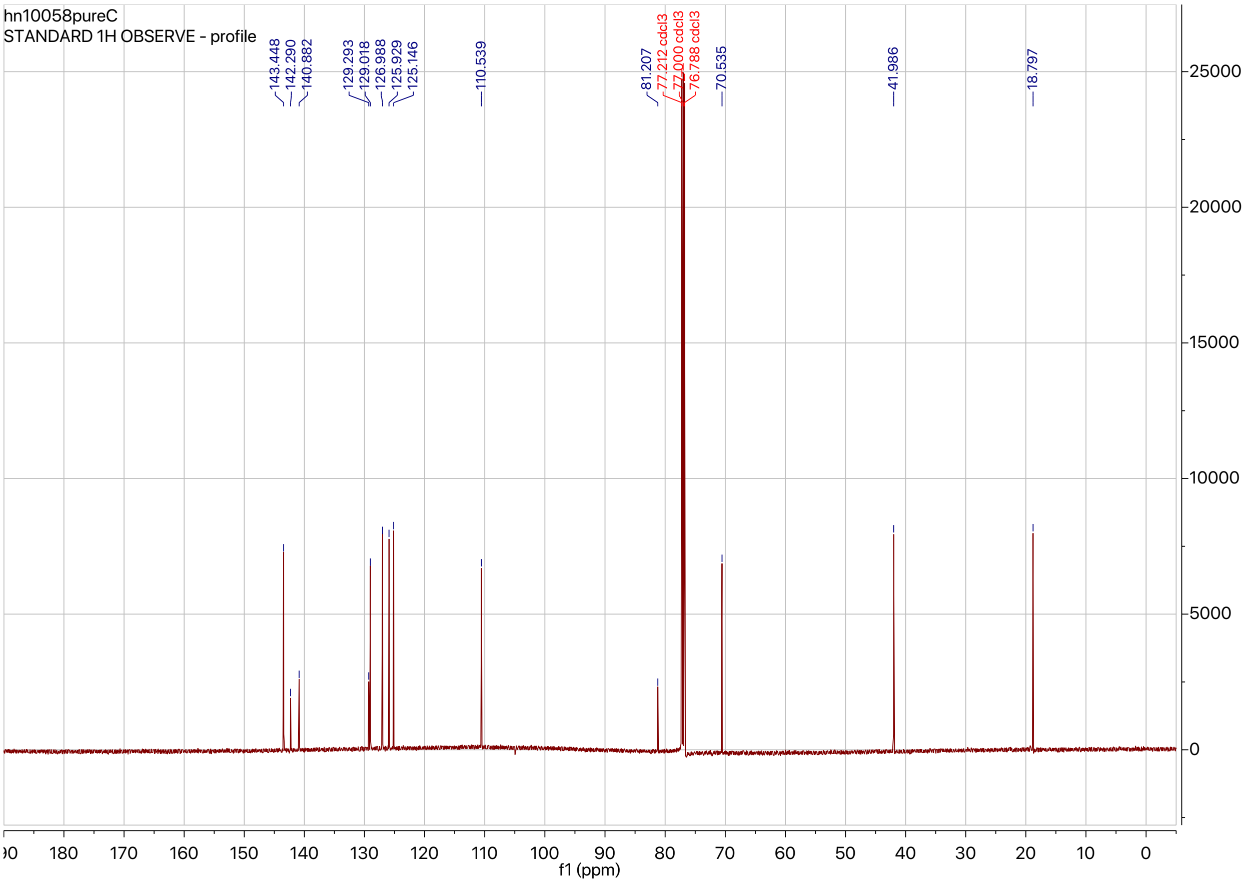

Supplement: Supplementary file 1 [file molecules-27-08701-s001.zip › molecules-2067259-supplementary.pdf]
